# Effects of Nutritional Supplements Alone or as an Adjunct to Nonsurgical Periodontal Therapy: A Systematic Review and Network Meta‐Analysis

**DOI:** 10.1155/ijod/4249289

**Published:** 2026-02-23

**Authors:** Haonuo Tang, Ruiqi Liang, Yiwen Chen, Chenyang Suo, Yong Chen

**Affiliations:** ^1^ Department of Stomatology, School of Medicine, Xiamen University, Xiamen, Fujian, 361000, China, xmu.edu.cn; ^2^ Department of Preventive Medicine, School of Public Health, Xiamen University, Xiamen, Fujian, 361000, China, xmu.edu.cn

**Keywords:** dentistry, network meta-analysis, nutritional supplements, periodontitis, periodontology, randomized controlled trials

## Abstract

**Background:**

Nutritional supplements (NSs) have been introduced as an adjunct to nonsurgical periodontal therapy (NSPT) in recent years. The aim of the present study was to evaluate the clinical effectiveness of NSs as an adjunct to NSPT by a network meta‐analysis (NMA).

**Methods:**

This study followed the PRISMA guidelines, and its protocol was registered with PROSPERO. We searched PubMed, Embase, Web of Science, and Cochrane Library for relevant studies. Gingival index (GI), plaque index (PI), probing pocket depth (PPD), clinical attachment level (CAL), and bleeding on probing (BOP) were selected as outcomes. A comparative analysis of different interventions was performed by using a Bayesian NMA model. Quality assessment was performed using the RoB2.0 tool.

**Results:**

Seventy‐nine studies were included, with seven types of NSs. Herbal antioxidants were the most effective in reducing BOP. VD and antioxidants + scaling and root planing (SRP) exhibited significant efficacy on alleviating CAL at 3‐month (3 m) and 6‐month (6 m) follow‐up. Additionally, herbal‐antioxidants + SRP, probiotic + SRP, and melatonin demonstrated good efficacy on ameliorating PI, GI, and PPD.

**Conclusions:**

Among the seven types of NSs identified in this NMA, some NSs exhibit relatively higher periodontal health benefits. However, evidence regarding the effectiveness of NSs as an adjunct to NSPT is still insufficient and warrants further high‐quality clinical trials.

## 1. Background

Periodontitis (PD) is a plaque‐associated chronic multifactorial inflammatory disease characterized by progressive destruction of periodontal supporting tissues [1]. Nonsurgical periodontal therapy (NSPT) is efficacious in removing plaque and relieving inflammation, while scaling and root planing (SRP) eliminates or reduces the presumed pathogens and transfers the microbiota to a more favorable environment for creating stable periodontal conditions [2–5].

In recent years, numerous studies have focused on the effectiveness of nutritional supplements (NSs) such as vitamins, minerals, and probiotics as an adjunct to NSPT in improving periodontal health and treating PD [6–9]. Research has demonstrated the clinical benefits of vitamins in ameliorating periodontal destruction [10, 11], the ability of some minerals to reduce the accumulation of dental plaque [12], and the antioxidative stress and immune‐improving effects of antioxidants [13, 14]. Vitamins (e.g., VC and VD) achieve antioxidant effects by neutralizing free radicals, immunomodulatory effects by regulating inflammatory mediators, and connective tissue‐related benefits by facilitating collagen synthesis and bone health [15]. With antimicrobial effects, minerals (e.g., zinc and magnesium) reduce dental plaque accumulation, support immune function, and contribute to tissue repair [12, 16]. In addition, herbal extracts as NSs can resist inflammation and improve periodontal parameters [16, 17], melatonin may be effective in antioxidative stress and PD treatment [18, 19], probiotics may be effective in inhibiting periodontal pathogens and assisting PD treatment [20, 21], and multinutrients may mitigate gingival inflammation and achieve an antimicrobial effect [22, 23]. These studies suggest that NSs with anti‐inflammatory and antioxidant effects may become a new adjunctive therapy to NSPT for PD patients.

The efficacy of vitamins, probiotics, and antioxidants as NSs on PD has been mostly explored in meta‐analyses. For example, Gheisary et al. [24] found in a meta‐analysis that probiotics can reduce the counts of subgingival periodontal pathogens such as *P. gingivalis*, *F. nucleatum*, and *T. forsythia*. Tada et al. [15] demonstrated the positive role of VC in preventing the incidence and progression of periodontal disease. Besides, López‐Valverde et al. [25] argued that antioxidants regulate some clinical periodontal parameters and inflammatory biomarkers. Despite numerous clinical trials and meta‐analyses on the effectiveness of single types of NSs, there has been no systematic analysis of the clinical benefits of different types of NSs.

Network meta‐analysis (NMA) is a weighted pooled methodology that combines direct and indirect evidence from all studies on multiple interventions [26, 27]. This NMA aimed to systematically assess and compare the clinical effectiveness of NSs as an adjunct to NSPT.

## 2. Materials and Methods

### 2.1. Registration

This NMA was conducted following PRISMA [28] and was registered with PROSPERO (CRD42024589010, https://www.crd.york.ac.uk/PROSPERO/).

### 2.2. Search Strategy

We extensively searched PubMed, Embase, Web of Science, and Cochrane Library for relevant studies from inception to July 24, 2024. The search terms included “periodontal disease,” “dietary supplement,” “antioxidant,” “melatonin,” “herbal extract,” “minerals,” and “vitamin.” In addition, we manually searched references in other relevant studies as well as gray literature for eligible studies. The specific search strategy is described in Supporting Information: Table S1.

#### 2.2.1. Inclusion Criteria

Population (P): Patients who were diagnosed with chronic PD by clinical examination, of any gender and age, with or without other diseases (such as diabetes).

Intervention (I): Various types of NSs (melatonin, antioxidants, multinutrients, probiotics, herbal extracts, minerals, and vitamins) in the test group, with varying dosages and duration. Specifically, herbal extracts were further categorized based on their main ingredients into herbal antioxidants and herbal minerals, while vitamins were also subdivided into VB, VD, and VC. When different concentrations of the same supplement were administered in one experiment, the higher concentration was selected to assess its maximum potential efficacy. In this way, the dose–response relation in PD adjunctive therapy could be better reflected, and efficacy could be less underestimated.

Comparator (C): Placebo, any NSs, SRP alone, or NSs + SRP.

Outcome (O): Periodontal parameters, including gingival index (GI), plaque index (PI), probing pocket depth (PPD), clinical attachment level (CAL), and bleeding on probing (BOP) at 3‐month (3 m) and 6‐month (6 m) follow‐up (or the closest time point if not). To reduce bias from baseline differences, all outcome metrics were analyzed as changes from baseline (Δ values).

Study design (S): Randomized controlled trials (RCTs).

Only English‐language studies were included.

#### 2.2.2. Exclusion Criteria

(1) Duplicate publications or duplicate study populations. (2) Non‐RCTs. (3) Studies using interventions other than NSPT, such as surgical periodontal therapies (e.g., flap surgery), laser‐assisted therapy, or other invasive procedures not based on SRP. (4) Animal studies. (5) Studies with the outcomes excluding the periodontal parameters listed above. (6) Studies lacking complete data.

#### 2.2.3. Data Extraction

Two authors (Tang and Liang) were independently responsible for study screening. First, studies were initially screened by reading the titles and abstracts. After the ineligible studies were excluded, the full text of the remaining studies was further examined to finally include the eligible ones. Any disagreement was resolved by discussion between the two authors or consultation with a third author (Suo) when necessary. The following critical data were extracted and recorded independently by the two authors using a pre‐established standardized spreadsheet: title, first author, year of publication, region, sample size, sex ratio, age, interventions, course of treatment, and outcomes (GI, PI, PPD, CAL, and BOP). All data extracted were entered into Excel to ensure accuracy.

#### 2.2.4. Quality Evaluation

The quality of included studies was evaluated by two authors (Suo and Chen) independently using the Cochrane Risk of Bias 2.0 (RoB2.0) [29] from the domains of random sequence generation, allocation concealment, blinding, incomplete difference data, and selective reporting. Each domain was rated as “high risk,” “some concerns,” or “low risk.” Specifically, the RoB was rated as low if the risk was low in all five domains or it was medium in only one domain and low in the rest; the RoB was rated as high if the risk was medium in four or more of the five domains or high in any one. The two authors independently assessed the RoB strictly according to the RoB2.0 criteria. Any disagreement was resolved by a third author (Tang). Inter‐rater reliability was also evaluated by a consistency test, and the result revealed substantial agreement between the two authors (kappa = 0.924).

#### 2.2.5. Statistical Analysis

The effect size was measured by weighted mean difference (WMD) or relative risk (RR) according to the form of baseline‐to‐outcome change (Δ values) in different studies. A Bayesian NMA model was established by the Markov chain Monte Carlo method, and iterated to estimate the relative efficacy of different interventions [30]. The testing process was performed with a model chain value of 4, an annealing value of 10,000, an iteration value of 50,000, a step size of 10 for each test, and an initial value of 2.5 to obtain the posterior distribution. Consistency and inconsistency models were established using the R gemtc package and assessed for the fit degree by comparing the deviance information criterion (DIC) value. A variation in DIC <5 between the two models was considered a successful model fit [31, 32]. The DIC value corresponding to each parameter is shown in Supporting Information: Table S2. The node‐splitting method was used for inconsistency tests between the direct and indirect evidence [31]. The results of the inconsistency test are presented in the Supporting file (Supporting Information: Figure S1–S3). *p*  > 0.05 indicated no inconsistency between direct and indirect comparisons. Moreover, a network diagram was plotted for the comparison of interventions, in which each node represented an intervention, its size represented the entire sample size, the connecting lines represented direct comparisons between two interventions, and the line thickness corresponded to the number of studies [33]. Finally, the interventions were ranked by calculating the surface under the cumulative ranking curve (SUCRA), with higher values indicating better intervention effects [34]. In addition, possible publication bias was assessed using funnel plots [35, 36], thereby guaranteeing the fairness and comprehensiveness of the analysis results. Finally, to investigate whether the mode of administration, sample size, or year of publication influenced the efficacy of different interventions, a meta‐regression analysis was performed using the R [37]. R4.41 and STATA18.0 were adopted for statistical analyses. *p*  < 0.05 suggested statistically significant.

## 3. Results

### 3.1. Search Results

A total of 32,422 studies were retrieved from the databases. After 4696 duplicate publications were removed using EndNote, 27,502 studies were excluded by reading the title and abstract. Then the remaining 224 studies were further screened for study populations, study design, interventions, and outcomes. Finally, 79 eligible studies were included after six studies were excluded due to unavailable full text, 36 due to non‐RCTs, 31 due to nonmatched interventions and populations, 13 due to nonchronic PD patients, and 58 due to no relevant outcomes or nonextractable data. The specific search strategy is shown in Supporting Information: Figure S4.

A total of seven different types of NSs were included, including herbal extracts in 34 studies, antioxidants in 18 studies, probiotics in 10 studies, minerals in two studies, vitamins in five studies, multinutrients in four studies, and melatonin in six studies. The complete reference list for all included studies is provided in Supporting Information: File S1.

### 3.2. Basic Characteristics and Quality Evaluation Results

Supporting Information: Table S3 shows the baseline information of the included studies. According to the study by Tonetti et al. [38], patients with chronic PD were categorized into Types I–III. Among the 79 RCTs included, approximately 3709 subjects aged 11–79 years were covered, with a male/female ratio of about 47.1%/52.9%. A total of thirty‐three studies were conducted in India, 10 in Iran, six in Egypt, and the rest in China, the United States, and Italy.

The RoB was rated as high in 15 studies (19%), low in 55 studies (69.6%), and some concern in nine studies (11.4%) (Supporting Information: Figure S5).

### 3.3. NMA Results

#### 3.3.1. Network Diagram

Each node represented one intervention, and its size was positively correlated with the relevant sample size, that is, the larger the node, the more the included studies. The connecting lines represented direct comparisons between two interventions, and the line thickness was positively correlated with the number of relevant studies, that is, the thicker the line, the more the included studies (Figure 1). The potential scale reduction factor (PSRF) was calculated for each dataset, and the closer the value is to 1, the better the convergence of the model.

Figure 1Network diagram of outcomes: (a) BOP; (b) CAL (3 m); (c) CAL (6 m); (d) GI; (e) PI; (f) PPD. anti, antioxidants; her‐anti, herbal extracts (antioxidants); her‐min, herbal extracts (minerals); mel, melatonin; min, minerals; mul, multinutrients; pro, probiotics; SRP, scaling and root planing.

**Figure:**
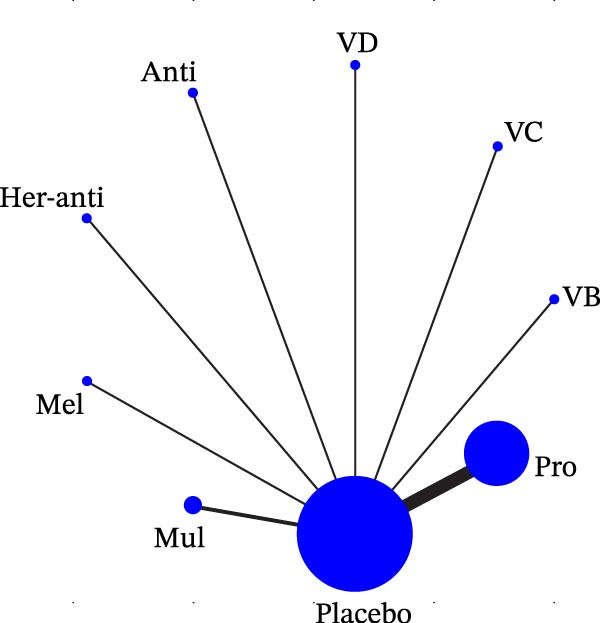
(a)

**Figure:**
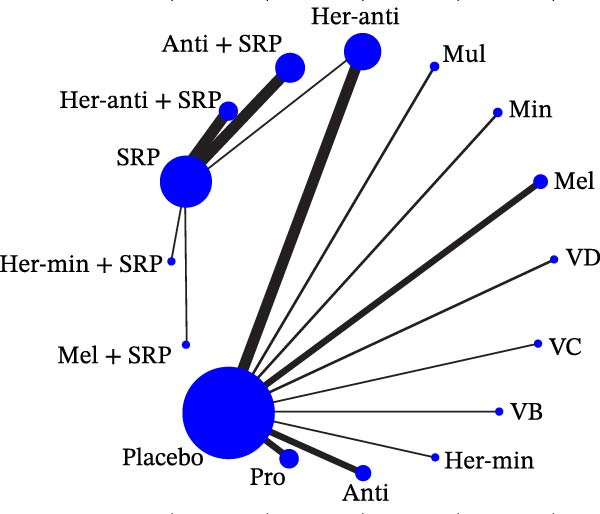
(b)

**Figure:**
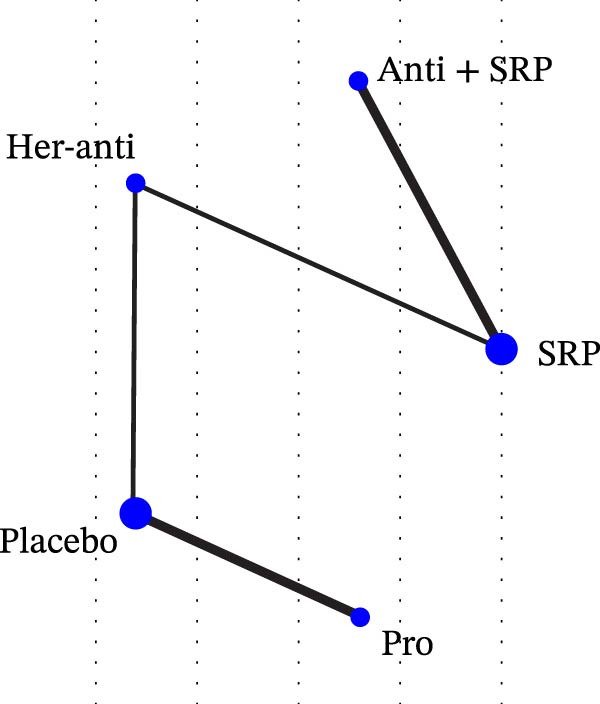
(c)

**Figure:**
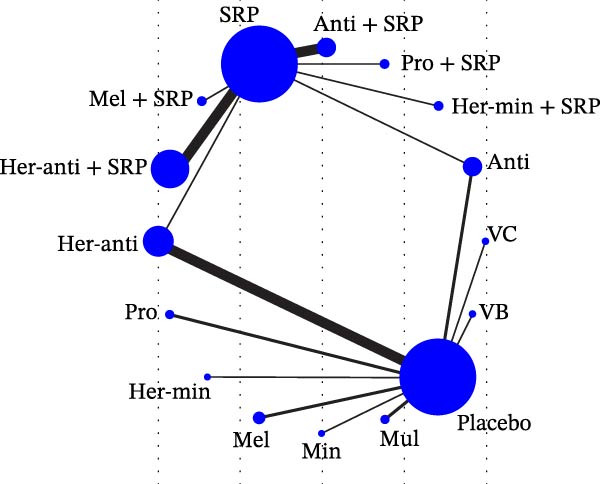
(d)

**Figure:**
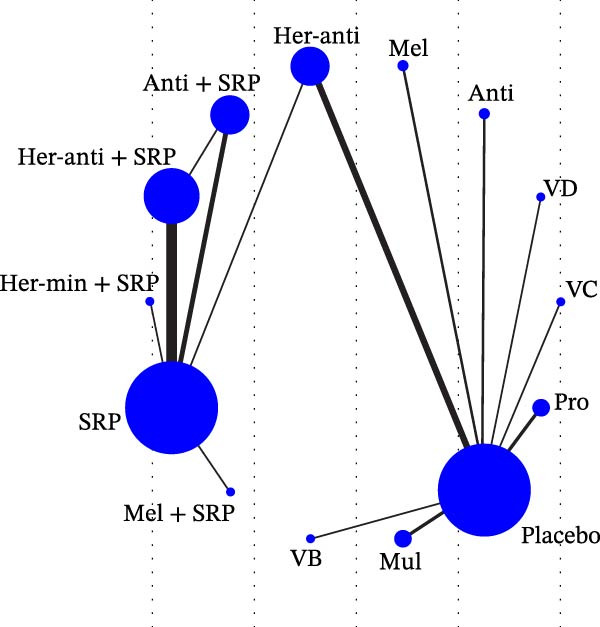
(e)

**Figure:**
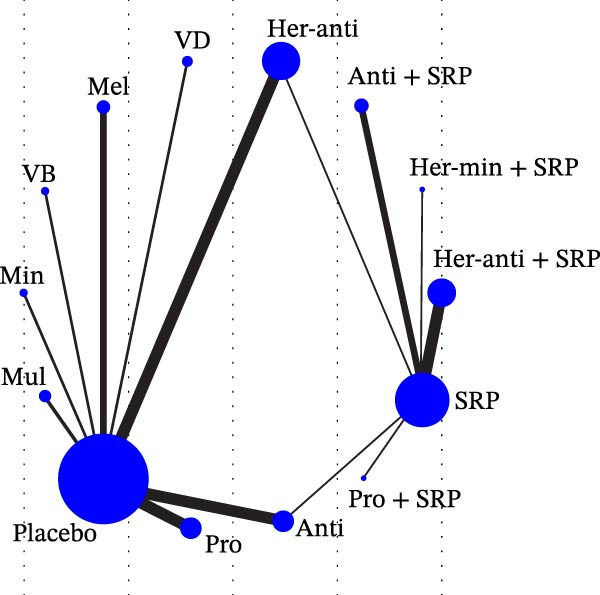
(f)

#### 3.3.2. BOP

Seventeen RCTs reported BOP. The NMA results showed that BOP significantly decreased after treatment with herbal antioxidants compared with placebo, multinutrients, VB, VD, VC, probiotics, melatonin, and antioxidants alone (herbal antioxidants vs. placebo: MD = 16.06, 95% CI = −10.27 to 42.35). To sum up, herbal antioxidants are superior to other interventions in improving BOP in PD, with statistically significant differences (*p*  < 0.05) (Supporting Information: Table S4). The forest plot for different NSs vs. placebo in improving BOP is presented in Supporting Information: Figure S6.

The SUCRA value was the highest for herbal antioxidants (76.99%), followed by multinutrients (70.3%) and melatonin (59.79%).

#### 3.3.3. CAL

The changes in CAL (3 m) and CAL (6 m) were followed up in 56 and 6 studies, respectively. For CAL (3 m), the NMA results showed that CAL significantly decreased after treatment with VD compared with placebo, herbal antioxidants, multinutrients, VB, VC, probiotics, melatonin, minerals, antioxidants, SRP, herbal minerals, herbal antioxidants + SRP, melatonin + SRP, and antioxidants + SRP (VD vs. placebo: MD = 1.23, 95% CI = 0.32–2.16; VD vs. probiotics: MD = 1.12, 95% CI = 0.05–2.21). To sum up, VD is superior to other interventions in improving CAL (3 m) in PD, with statistically significant differences (*p*  < 0.05). For CAL (6 m), the NMA results showed that CAL significantly decreased after treatment with antioxidants + SRP compared with placebo, herbal antioxidants, probiotics, and SRP (antioxidants + SRP vs. placebo: MD = 0.91, 95% CI = −1.67 to 3.61). The league tables for improvements in CAL (3 m) and CAL (6 m) are shown in Supporting Information: Tables S5 and S6, respectively, and the corresponding forest plot is shown in Supporting Information: Figure S7.

For CAL (3 m), the SUCRA value was the highest for VD (87.32%), followed by melatonin (81.21%) and melatonin + SRP (69.98%). For CAL (6 m), the SUCRA value was the highest for antioxidants + SRP (79.23%), followed by herbal antioxidants (67.18%) and SRP (43.8%).

#### 3.3.4. GI

GI was reported in 43 studies. The NMA results revealed that probiotic + SRP significantly reduced GI as compared to placebo, multinutrients, VC, VB, probiotics, melatonin, antioxidants, minerals, SRP, herbal antioxidants, herbal minerals, herbal antioxidants + SRP, herbal minerals + SRP, melatonin + SRP, and antioxidants + SRP (probiotic + SRP vs. antioxidants: MD = 1.02, 95% CI = 0.01–2.04; probiotic + SRP vs. SRP: MD = 0.84, 95% CI = 0.02–1.66). To sum up, probiotic + SRP is more effective in improving GI in PD than other interventions, with statistically significant differences (*p*  < 0.05) (Supporting Information: Table S7). The forest plot for different NSs vs. placebo in improving GI is displayed in Supporting Information: Figure S8.

The SUCRA value was the highest for probiotic + SRP (85.08%), followed by mineral (78.7%) and herbal antioxidants (68.27%).

#### 3.3.5. PI

PI was reported in 48 RCTs. The NMA results revealed that herbal antioxidants + SRP significantly reduced PI as compared to placebo, herbal antioxidants, multinutrients, VC, VB, VD, probiotics, antioxidants, melatonin, herbal minerals + SRP, SRP, melatonin + SRP, and antioxidants + SRP (herbal antioxidants + SRP vs. SRP: MD = 0.27, 95% CI = 0.09–0.44). To sum up, herbal antioxidants + SRP is more effective in improving PI in PD than other interventions, with statistically significant differences (*p*  < 0.05) (Supporting Information: Table S8). The forest plot is shown in Supporting Information: Figure S9.

The SUCRA value was the highest for herbal antioxidants + SRP (80.58%), followed by antioxidants (67.75%) and antioxidants + SRP (63.12%).

#### 3.3.6. PPD

The changes in PPD (3 m) were followed up in 65 studies. The NMA results revealed that melatonin significantly reduced PPD as compared to placebo, herbal antioxidants, multinutrients, VB, VD, probiotics, minerals, antioxidants, SRP, probiotic + SRP, antioxidants + SRP, herbal antioxidants + SRP, and herbal minerals + SRP (melatonin vs. placebo: MD = 1.11, 95% CI = 0.49–1.74; melatonin vs. probiotics: MD = 1.00, 95% CI = 0.23–1.77). To sum up, melatonin is more effective in improving PPD (3 m) than other interventions, with statistically significant differences (*p*  < 0.05) (Supporting Information: Table S9). Since no loop was formed in the network diagram, the PPD (6 m) follow‐up data were ultimately deleted. The forest plot is shown in Supporting Information: Figure S10.

For PPD (3 m), the SUCRA value was the highest for melatonin (78.12%), followed by herbal minerals + SRP (75.65%) and probiotic + SRP (75.53%).

SUCRA for different outcomes in different interventions is shown in Figure 2.

Figure 2(a) BOP; (b) CAL (3 m); (c) CAL (6 m); (d) GI; (e) PI; (f) PPD. anti, antioxidants; her‐anti, herbal extracts (antioxidants); her‐min, herbal extracts (minerals); mel, melatonin; min, minerals; mul, multinutrients; pro, probiotics; SRP, scaling and root planing.

**Figure:**
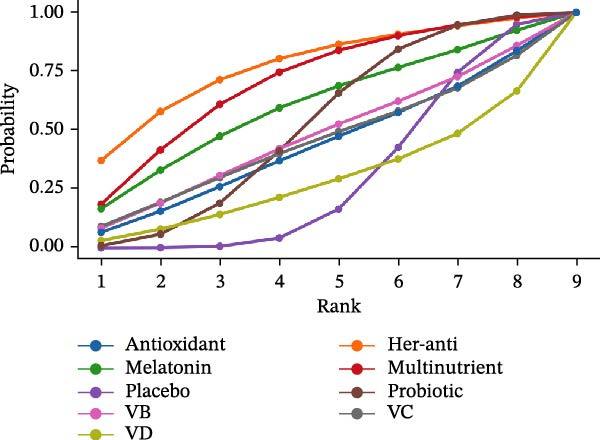
(a)

**Figure:**
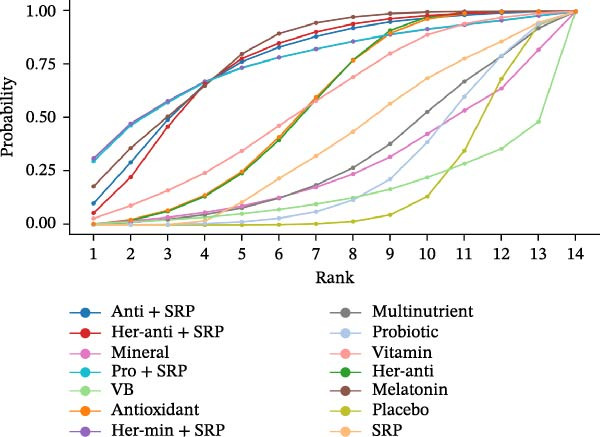
(b)

**Figure:**
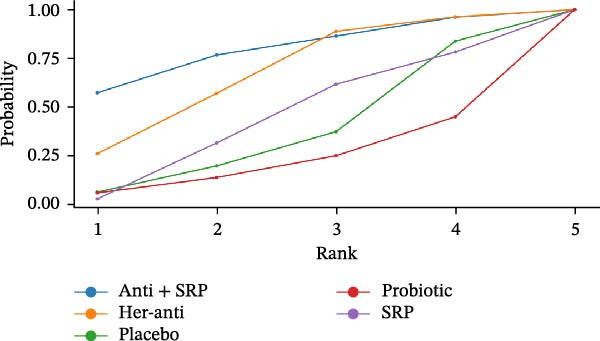
(c)

**Figure:**
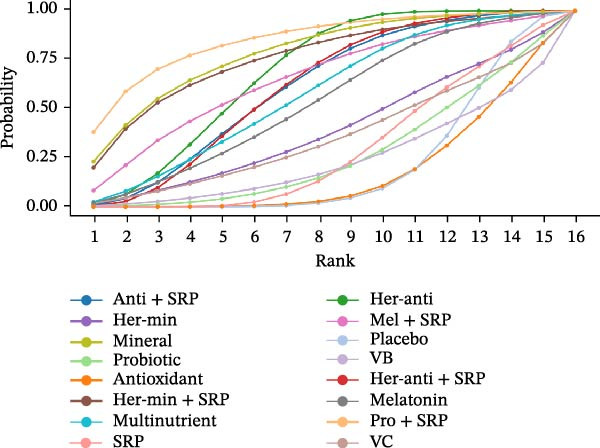
(d)

**Figure:**
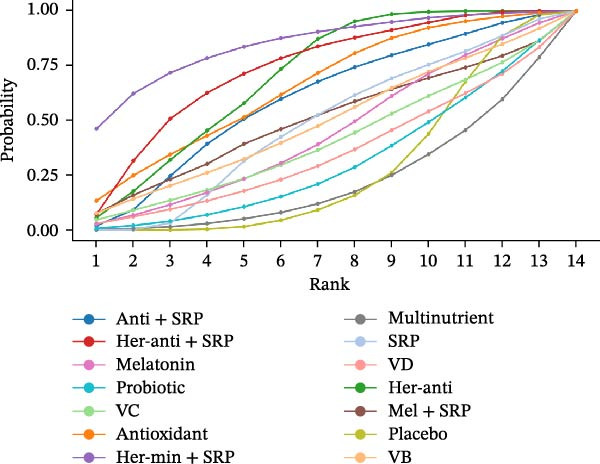
(e)

**Figure:**
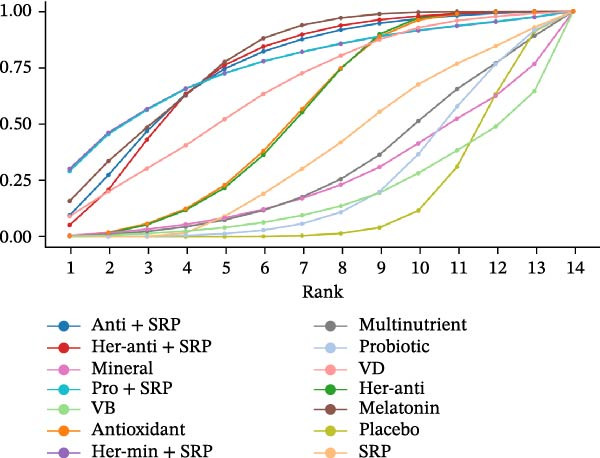
(f)

#### 3.3.7. Publication Bias

The comparison‐adjusted funnel plot was drawn for the publication bias. It was found that the plot was roughly left–right symmetric, suggesting a low possibility of publication bias (Supporting Information: Figure S11).

#### 3.3.8. Meta‐Regression Analysis Results

Meta‐regression analyses were conducted on year of publication, sample size, and mode of administration in each study. The results showed that the year of publication and sample size had no significant influence. The mode of administration might influence BOP and PI rather than CAL, PPD, and GI (*p*  < 0.05).

## 4. Discussion

This study compared the efficacy of various NSs (herbal extracts, antioxidants, melatonin, minerals, and vitamins) on PD for the first time. Previous studies have shown that NSs contain energy, protein, multivitamins, and minerals [39], so they possess many clinical benefits in oral health [40–42]. Previous meta‐analyses on NSPT focused mostly on the efficacy of vitamins or probiotics on PD [15, 25]. Therefore, this NMA intends to compare several different types of NSs, hoping to provide the best evidence for clinical treatment.

### 4.1. Herbal Extracts

The herbal extracts herein referred to NSs are primarily composed of plants and their processed extracts, as well as some synthesized chemical components derived from them. Nowadays, herbal extracts have been applied mainly in the form of mouthwashes in clinical settings [43]. Given an excessively broad scope of herbal extracts, they were categorized based on their primary mechanisms into herbal antioxidants and herbal minerals. In this NMA, herbal antioxidants alone achieved the best but not significant efficacy on BOP, while herbal antioxidants + SRP achieved a significant efficacy on PI. The cleansing, astringent, and antimicrobial effects of herbal extracts have been demonstrated in many studies [16, 44, 45], suggesting the potential of herbal extracts in treating PD. Due to differences in their main ingredients, the efficacy of herbal extracts may vary in PD treatment. Varma et al. showed that a herbal extract known as tea tree oil (TTO) can dissolve pus‐producing clumps of leukocytes in the bloodstream and thus facilitate the removal of infections such as scabies and ulcers [46], suggesting that herbal antioxidants may achieve antioxidant and anti‐inflammatory effects in PD treatment. Varoni et al. [17] argued that some of the herbal extracts have anticaries, PD, and candidiasis activity. The above studies verified the findings of this study that herbal extracts were significantly efficacious in treating periodontal diseases. However, since different herbal extracts or mixed herbal extracts were used in the clinical trials included, evidence proving the effectiveness of herbal extracts as an adjunct to NSPT is still insufficient. Additionally, herbal extracts cannot be simply represented by a single ingredient, so their ingredients should be categorized in more detail to determine which ingredient has the optimal efficacy on PD.

### 4.2. Probiotics

The efficacy of probiotics as an adjunct to NSPT has been widely discussed in previous meta‐analyses [21, 47]. This study revealed that probiotic + SRP was the most effective in improving GI. Probiotics may restore the oral microbial ecological balance by competitively inhibiting periodontal pathogens and increasing beneficial bacteria [20]. Mendonça et al. [21] discussed the efficacy of different types of probiotic + SRP in treating PD in an NMA, and found that most types of probiotic + SRP are more effective in improving PPD and CAL. Gheisary et al. [24] showed in a meta‐analysis that probiotics can reduce the counts of subgingival periodontal pathogens. However, long‐term results are still lacking for microbial outcomes of probiotic + SRP [47, 48], so the evidence indicating that probiotics have clinical periodontal benefits is still not sufficient.

### 4.3. Melatonin

We found that melatonin possessed great advantages in reducing PPD. Melatonin may play a key role in protecting tissues from oxidative stress injury [18], and it is more protective than other antioxidants in vivo [49]. Sethi et al. argued that due to its capability to regulate osteocytes, resist oxidation, and facilitate angiogenesis, melatonin is superior in bone healing [50], which also provides a theoretical basis for the use of melatonin in NSPT. Corbella et al. [19] discovered that melatonin can reduce PPD in systemically healthy patients, but the quality of evidence was low. Another review conveyed that melatonin may have a positive effect in the treatment of PD, but the overall quality of available evidence was too low to verify the effectiveness of melatonin as an adjunct to NSPT [51].

### 4.4. Minerals

In this study, the role of minerals in NSPT was not well defined. The mineral content in the body is closely related to the development of PD, and PD patients with diabetes mellitus suffer from a significant imbalance of mineral elements [52]. Therefore, mineral supplements may help to relieve inflammation in PD. Fatima [12] confirmed that zinc‐containing mouthwashes and toothpastes can maintain high zinc concentrations in plaque and saliva to control plaque formation and suppress tartar formation. In the RCTs included, Jayachandran et al. studied the role of calcium supplementation in PD treatment, and found that calcium may alleviate PD symptoms by facilitating bone formation [53]. However, minerals function predominantly in the surgical treatment of PD [54], so the types of minerals working in NSPT remained limited in this study, as illustrated in the network diagram. Therefore, more high‐quality studies are needed to investigate the effects of minerals as an adjunct to NSPT.

### 4.5. Multinutrients

This study revealed certain clinical benefits of multinutrients as NSs on PD in some respects, but their effect was not significant. Laky et al. [55] found in a clinical study that multinutrient supplements are useful in improving BOP and PPD, which, however, have no statistically significant difference from placebo, consistent with the findings of this study. Therefore, more clinical studies are required to investigate the clinical efficacy of multinutrients.

### 4.6. Vitamins

In this study, VD exhibited certain advantages in improving CAL (3 m), while other vitamins showed no significant efficacy on PD. Consistent with our findings, Gao et al. [56] found that VD improves CAL in PD patients through its effects on bone metabolism and inflammation regulation. Additionally, Tada et al. [15] demonstrated the good effect of VC on alleviating PD progression. According to the available results, vitamins can increase collagen synthesis and promote bone metabolism in periodontal tissue repair and regeneration [10, 11], which may produce some clinical benefits. However, VD was used as an NS for treating PD in only one study, so significant publication bias might exist. Therefore, more studies are required to verify the efficacy and stability of vitamins.

### 4.7. Antioxidants

In this study, antioxidants + SRP exhibited certain but not significant advantages in improving CAL (6 m). As shown in the existing literature, oxidative stress weakens the antioxidant defense response during the periodontal destruction process [13], providing a feasibility for the use of antioxidants in the treatment of PD. Research suggests that antioxidants such as omega‐3 PUFAs and their ester derivatives display potent antibacterial activity against a variety of oral pathogens, such as *Candida albicans* and periodontal pathogens, also indicating their certain antibacterial capability. A meta‐analysis by Castro et al. revealed the effectiveness of antioxidants as an adjunct to NSPT, consistent with the results of this study. However, outcomes may vary across modes of administration and types of antioxidants [57]. Due to high heterogeneity in the ingredients, dosage, and mode of administration, as well as short follow‐up periods, more evidence is required for the long‐term efficacy and optimal regimen of antioxidants.

### 4.8. Meta‐Regression

Meta‐regression analysis revealed that the mode of administration possibly influenced both PI and BOP. The possible reason is that mouthwashes may more easily enter inflamed tissues to remove plaque and stop bleeding, which are potentially more effective in improving PI and BOP in localized treatment. To address this potential bias, the mode of administration will be further categorized in the future to reduce outcome bias arising from different routes of administration.

This NMA demonstrated clinical benefits of some NSs, such as probiotics, herbal extracts, and melatonin, in NSPT, consistent with previous meta‐analyses [15, 19, 24]. However, this NMA for the first time ranked the relative efficacy of multiple NSs, offering a valuable reference for clinical selection. In the future, long‐term, high‐quality RCTs are required, and the optimal dosing strategies and personalized regimens should be explored. Additionally, NSs can be combined with other adjunctive therapies, such as ozone therapy [58] and photobiomodulation [59], to assess the potential synergistic effects. In this way, a more effective adjunct to NSPT can be developed and warrants further validation.

This study has several strengths. First, the effects of different types of NSs on periodontal parameters in PD patients were studied for the first time, and a large number of studies were included. By pooling study data, more comprehensive and credible results could be obtained. Second, a Bayesian NMA model was used for simultaneous comparison of interventions, which could help identify the optimal interventions and provide a new direction for the clinical treatment of PD [60, 61].

Finally, there are some limitations in this study. Firstly, some of the findings still need to be validated with more clinical evidence due to an inadequate number of included studies related to some of the interventions and a lack of clinical trials. Secondly, studies with high RoB due to no blinding or excessive loss to follow‐up were not excluded, which might influence the results. Additionally, reliance on baseline change was another potential limitation of this study. Although Δ values were used, residual bias might also be introduced if substantial variability was present in baseline periodontal parameters across studies. Future studies should standardize baseline assessments to enhance comparability.

## 5. Conclusion

Among the seven types of NSs identified in this NMA, herbal extracts, probiotics, melatonin, vitamins, and antioxidants achieve relatively higher periodontal health benefits, and when combined with SRP, they often yield greater clinical benefits. However, due to the limited available data, evidence for these NSs as an adjunct to NSPT remains insufficient. Therefore, their effectiveness still needs exploration by more high‐quality clinical studies.

**Nomenclature**
NSs: Nutritional supplements
NSPT: Nonsurgical periodontal therapy
NMA: Network meta‐analysis
GI: Gingival index
PI: Plaque index
PPD: Probing pocket depth
CAL: Clinical attachment loss
BOP: Bleeding on probing
SRP: Scaling and root planning
SUCRA: Surface under the cumulative ranking curve
PD: Periodontitis
RCT: Randomized controlled trials.

## Author Contributions

All authors contributed to the study conception and design. **Haonuo Tang:** conceptualization, methodology, software, formal analysis, data curation, resources, writing – original draft, writing – review and editing. **Ruiqi Liang:** conceptualization, methodology, software, formal analysis, data curation, writing – original draft, writing – review and editing. **Yiwen Chen:** resources, investigation, validation, formal analysis, writing – original draft, writing – review and editing. **Chenyang Suo:** resources, investigation, validation, formal analysis, writing – original draft, writing – review and editing. **Yong Chen:** conceptualization, methodology, validation, writing – review and editing, visualization, supervision, project administration.

## Funding

The authors declare that no funds, grants, or other support were received during the preparation of this manuscript.

## Disclosure

All authors read and approved the final manuscript.

## Ethics Statement

As this was a systematic review and meta‐analysis, ethical approval was not necessary.

## Consent

The authors have nothing to report.

## Conflicts of Interest

The authors declare no conflicts of interest.

## Supporting Information

Additional supporting information can be found online in the Supporting Information section.

## Supporting information


**Supporting Information** File S1: Complete Reference List of 79 Included Studies Categorized by Nutritional Supplement Type. Figure S1: The result of the inconsistency test for GI. Figure S2: The result of the inconsistency test for PI. Figure S3: The result of the inconsistency test for PPD. Figure S4: PRISMA® flow diagram of study search and results. Figure S5: RoB assessment result. Figure S6: Forest plot for the decline in BOP across interventions. Figure S7: Forest plot for the decline in CAL across interventions. A‐3m; B‐6m. Figure S8: Forest plot for the decline in GI across interventions. Figure S9: Forest plot for the decline in PI across interventions. Figure S10: Forest plot for the decline in PPD across interventions. Figure S11: Funnel plots for different outcomes: (a) BOP; (b) CAL (3m); (c) CAL (6m); (d) GI; (e) PI; (f) PPD. Table S1: Specific search strategy for PubMed. Table S2: DIC of different periodontal parameters. Table S3: Overview of included studies in this NMA. Table S4: NMA results for the decline in BOP across interventions. Table S5: NMA results for the decline in CAL (3m) across interventions. Table S6: NMA results for the decline in CAL (6m) across interventions. Table S7: NMA results for the decline in GI across interventions. Table S8: NMA results for the decline in PI across interventions. Table S9: NMA results for the decline in PPD across interventions.

## Data Availability

The original contributions presented in the study are included in the article, further inquiries can be directed to the corresponding author.
