# Supplementary material for: Effects of Nutritional Supplements Alone or as an Adjunct to Nonsurgical Periodontal Therapy: A Systematic Review and Network Meta‐Analysis
Source: Int J Dent. 2026 Feb 23;2026:4249289. doi: 10.1155/ijod/4249289 (PMC12929180; doi:10.1155/ijod/4249289)
Supplement: Supplementary file 1 — Supporting Information File S1: Complete Reference List of 79 Included Studies Categorized by Nutritional Supplement Type. Figure S1: The result of the inconsistency test for GI. Figure S2: The result of the inconsistency test for PI. Figure S3: The result of the inconsistency test for PPD. Figure S4: PRISMA® flow diagram of study search and results. Figure S5: RoB assessment result. Figure S6: Forest plot for the decline in BOP across interventions. Figure S7: Forest plot for the decline in CAL across interventions. A‐3m; B‐6m. Figure S8: Forest plot for the decline in GI across interventions. Figure S9: Forest plot for the decline in PI across interventions. Figure S10: Forest plot for the decline in PPD across interventions. Figure S11: Funnel plots for different outcomes: (a) BOP; (b) CAL (3m); (c) CAL (6m); (d) GI; (e) PI; (f) PPD. Table S1: Specific search strategy for PubMed. Table S2: DIC of different periodontal parameters. Table S3: Overview of included studies in this NMA. Table S4: NMA results for the decline in BOP across interventions. Table S5: NMA results for the decline in CAL (3m) across interventions. Table S6: NMA results for the decline in CAL (6m) across interventions. Table S7: NMA results for the decline in GI across interventions. Table S8: NMA results for the decline in PI across interventions. Table S9: NMA results for the decline in PPD across interventions. [file IJOD-2026-4249289-s001.docx]

**File S1** Complete Reference List of 79 Included Studies Categorized by Nutritional Supplement Type.

1. Ali A, Saliem S, Abdulkareem A, Radhi H, Gul S. Evaluation of the efficacy of lycopene gel compared with minocycline hydrochloride microspheres as an adjunct to nonsurgical periodontal treatment: a randomised clinical trial. *Journal of dental sciences*. 2021;16(2):691‐699. https://doi.org/10.1016/j.jds.2020.09.009

2. Eldessouky HF, Marie M. Nonsurgical Treatment of Periodontitis in Menopausal Patients: A Randomized Control Trial. *Biomed Research International*. 2024;2024:6997142-Article No.: 6997142. https://doi.org/10.1155/2024/6997142

3. Elgendy EA, Kazem HH. Effect of Omega-3 Fatty Acids on Chronic Periodontitis Patients in Postmenopausal Women: A Randomised Controlled Clinical Study. *Oral health & preventive dentistry*. 2018;16(4):327-332. https://doi.org/10.3290/j.ohpd.a40957

4. Prasanth T, Singh H, Krishna A, et al. Clinico-immunological evaluation of use of omega-3 fatty acids as nutraceutical approach in management of patients with chronic periodontitis: a randomized clinical trial. *Armed Forces medical journal, India*. 2024;80(4):449-457. https://doi.org/10.1016/j.mjafi.2024.04.013

5. Abdallah Khalil A, Alaaeldin E. Sustained Release of Liposomal Curcumin: Enhanced Periodontal Outcomes in Diabetic Patients. *The Chinese journal of dental research*. 2024;27(2):169-174. https://doi.org/10.3290/j.cjdr.b5459607

6. Hong JY, Lee JS, Choi SH, et al. A randomized, double-blind, placebo-controlled multicenter study for evaluating the effects of fixed-dose combinations of vitamin C, vitamin E, lysozyme, and carbazochrome on gingival inflammation in chronic periodontitis patients. *BMC oral health*. 2019;19(1):40. https://doi.org/10.1186/s12903-019-0728-2

7. Acharya S, Gujjari SK, Murthy KS, Battula R. Evaluation of grape seed formulation as an adjunct to scaling and root planing on oxidative stress, inflammatory status and glycaemic control in Type-2 diabetic patients with chronic periodontitis: A randomised controlled trial. *Journal of Clinical and Diagnostic Research*. 2021;15(4):ZC20-ZC25. https://doi.org/10.7860/JCDR/2021/45235.14792

8. Agarwal A, Chaudhary B. Clinical and microbiological effects of 1% Matricaria chamomilla mouth rinse on chronic periodontitis: A double-blind randomized placebo controlled trial. *Journal of Indian Society of Periodontology*. 2020;24(4):354-361. https://doi.org/10.4103/jisp.jisp_441_19

9. Aslroosta H, Paknejad M, Davari M, Akbari S, Taheri M, Abdollahi M. Semelil as Adjunctive Therapy in Chronic Periodontitis: A Preliminary Randomized Controlled Clinical Study. *Iranian Journal of Pharmaceutical Research*. 2021;20(1):339-347. https://doi.org/10.22037/ijpr.2020.113604.14399

10. Babaei H, Forouzandeh F, Maghsoumi-Norouzabad L, Yousefimanesh HA, Ravanbakhsh M, Zare Javid A. Effects of Chicory Leaf Extract on Serum Oxidative Stress Markers, Lipid Profile and Periodontal Status in Patients With Chronic Periodontitis. *Journal of the American College of Nutrition*. 2018;37(6):479-486. https://doi.org/10.1080/07315724.2018.1437371

11. Borgohain R, Fatima N, Bagde H, Dhanai A, Mukherjee K, Joshi P. Efficacy of cucurmin and aloevera extracts gel as local drugs delivery agents. *Journal of Pharmacy and Bioallied Sciences*. 2023;15(6):S981-S983. https://doi.org/10.4103/jpbs.jpbs_259_23

12. Boyapati R, Gojja P, Chintalapani S, Nagubandi K, Ramisetti A, Salavadhi SS. Efficacy of local drug delivery of Achyranthes aspera gel in the management of chronic periodontitis: A clinical study. *Journal of Indian Society of Periodontology*. 2017;21(1):46-49. https://doi.org/10.4103/jisp.jisp_130_17

13. Chopra A, Thomas BS, Sivaraman K, Prasad HK, Kamath SU. Green Tea Intake as an Adjunct to Mechanical Periodontal Therapy for the Management of Mild to Moderate Chronic Periodontitis: a Randomized Controlled Clinical Trial. *Oral health & preventive dentistry*. 2016;14(4):293‐303. https://doi.org/10.3290/j.ohpd.a36100

14. Deore GD, Gurav AN, Patil R, Shete AR, Naiktari RS, Inamdar SP. Herbal anti-inflammatory immunomodulators as host modulators in chronic periodontitis patients: a randomised, double-blind, placebo-controlled, clinical trial. *Journal of periodontal & implant science*. 2014;44(2):71‐78. https://doi.org/10.5051/jpis.2014.44.2.71

15. Elavarasu S, Suthanthiran T, Thangavelu A, Alex S, Palanisamy VK, Kumar TS. Evaluation of superoxide dismutase levels in local drug delivery system containing 0.2% curcumin strip as an adjunct to scaling and root planing in chronic periodontitis: A clinical and biochemical study. *Journal of Pharmacy and Bioallied Sciences*. 2016;8:S48-S52. https://doi.org/10.4103/0975-7406.191967

16. Gholinezhad H, Bazyar H, Rashidi H, Salehi P, Haghighi-zadeh MH, Zare Javid A. Using ginger supplement in adjunct with non-surgical periodontal therapy improves metabolic and periodontal parameters in patients with type 2 diabetes mellitus and chronic periodontitis: a double-blind, placebo-controlled trial. *Journal of herbal medicine*. 2020;20:100315. https://doi.org/10.1016/j.hermed.2019.100315

17. Gottumukkala SNVS, Koneru S, Mannem S, Mandalapu N. Effectiveness of sub gingival irrigation of an indigenous 1% curcumin solution on clinical and microbiological parameters in chronic periodontitis patients: A pilot randomized clinical trial. *Contemporary clinical dentistry*. 2013;4(2):186-91. https://doi.org/10.4103/0976-237x.114874

18. Gunjal S, Hampiholi V, Ankola AV, Pateel DGS. Comparison of the effectiveness of Morus alba and chlorhexidine gels as an adjunct to scaling and root planing on stage II periodontitis - A randomized controlled clinical trial. *Int J Dent Hyg*. 2024;22(3):717-726. https://doi.org/10.1111/idh.12781

19. Javid AZ, Bazyar H, Gholinezhad H, et al. The effects of ginger supplementation on inflammatory, antioxidant, and periodontal parameters in type 2 diabetes mellitus patients with chronic periodontitis under non-surgical periodontal therapy. A double-blind, placebo-controlled trial. *Diabetes, metabolic syndrome and obesity*. 2019;12:1751‐1761. https://doi.org/10.2147/DMSO.S214333

20. Jünger H, Jaun-Ventrice A, Guldener K, Ramseier CA, Reissmann DR, Schimmel M. Anti-inflammatory potential of an essential oil-containing mouthwash in elderly subjects enrolled in supportive periodontal therapy: a 6-week randomised controlled clinical trial. *Clinical oral investigations*. 2020;24(9):3203-3211. https://doi.org/10.1007/s00784-019-03194-3

21. Kim Y-R, Nam S-H. Effect of oral gargle containing <i>Lespedeza cuneata</i> extract on periodontal health improvement and disease prevention: a randomized, controlled clinical trial. *Bmc Oral Health*. 2023;23(1)https://doi.org/10.1186/s12903-023-02816-3

22. Mahendra J, Mahendra L, Svedha P, Cherukuri S, Romanos GE. Clinical and microbiological efficacy of 4% Garcinia mangostana L. pericarp gel as local drug delivery in the treatment of chronic periodontitis: A randomized, controlled clinical trial. *Journal of investigative and clinical dentistry*. 2017;8(4):e12262. https://doi.org/10.1111/jicd.12262

23. Mohammad CA. Efficacy of curcumin gel on zinc, magnesium, copper, IL-1β, and TNF-α in chronic periodontitis patients. *BioMed research international*. 2020;2020:8850926. https://doi.org/10.1155/2020/8850926

24. Naiktari RS, Gaonkar P, Gurav AN, Khiste SV. A randomized clinical trial to evaluate and compare the efficacy of triphala mouthwash with 0.2% chlorhexidine in hospitalized patients with periodontal diseases. *Journal of periodontal & implant science*. 2014;44(3):134‐140. https://doi.org/10.5051/jpis.2014.44.3.134

25. Pappu R, Varghese J, Koteshwara KB, Kamath V, Lobo R, Nimmy K. Evaluation of biodegradable gel containing flax seed extract (Linum usitatissimum) as a targeted drug delivery for management of chronic periodontitis. *Journal of Herbal Medicine*. 2019;15:100254. https://doi.org/10.1016/j.hermed.2018.100254

26. Phogat M, Rana T, Prasad N, Baiju CS. Comparative evaluation of subgingivally delivered xanthan-based chlorhexidine gel and herbal extract gel in the treatment of chronic periodontitis. *Journal of Indian Society of Periodontology*. 2014;18(2):172-7. https://doi.org/10.4103/0972-124x.131319

27. Pradeep AR, Garg V, Raju A, Singh P. Adjunctive Local Delivery of Aloe Vera Gel in Patients With Type 2 Diabetes and Chronic Periodontitis: a Randomized, Controlled Clinical Trial. *Journal of periodontology*. 2016;87(3):268‐274. https://doi.org/10.1902/jop.2015.150161

28. Raghava KV, Sistla KP, Narayan SJ, Yadalam U, Bose A, Mitra K. Efficacy of Curcumin as an Adjunct to Scaling and Root Planing in Chronic Periodontitis Patients: A Randomized Controlled Clinical Trial. *The journal of contemporary dental practice*. 2019;20(7):842-846. https://doi.org/10.5005/jp-journals-10024-2608

29. Rahalkar A, Kumathalli K, Kumar R. Determination of efficacy of curcumin and Tulsi extracts as local drugs in periodontal pocket reduction: A clinical and microbiological study. *Journal of Indian Society of Periodontology*. 2021;25(3):197-202. https://doi.org/10.4103/jisp.jisp_158_20

30. Rayyan M, Terkawi T, Abdo H, et al. Efficacy of grape seed extract gel in the treatment of chronic periodontitis: A randomized clinical study. *Journal of investigative and clinical dentistry*. 2018;9(2):e12318. https://doi.org/10.1111/jicd.12318

31. Shirakawa S, Matsushima Y, Kajiyama S, et al. Effects of natural herb-containing dental toothpaste on periodontal pathogenic bacteria and clinical parameters: A randomized clinical trial. *Journal of Herbal Medicine*. 2021;30https://doi.org/10.1016/j.hermed.2021.100517

32. Singh A, Sridhar R, Shrihatti R, Mandloy A. Evaluation of Turmeric Chip Compared with Chlorhexidine Chip as a Local Drug Delivery Agent in the Treatment of Chronic Periodontitis: A Split Mouth Randomized Controlled Clinical Trial. *J Altern Complement Med*. 2018;24(1):76-84. https://doi.org/10.1089/acm.2017.0059

33. Taalab MR, Mahmoud SA, Moslemany RME, Abdelaziz DM. Intrapocket application of tea tree oil gel in the treatment of stage 2 periodontitis. *BMC oral health*. 2021;21(1):239. https://doi.org/10.1186/s12903-021-01588-y

34. Taleghani F, Rezvani G, Birjandi M, Valizadeh M. Impact of green tea intake on clinical improvement in chronic periodontitis: a randomized clinical trial. *Journal of stomatology, oral and maxillofacial surgery*. 2018;119(5):365‐368. https://doi.org/10.1016/j.jormas.2018.04.010

35. Theodoro LH, Cláudio MM, Nuernberg MAA, et al. Effects of Lactobacillus reuteri as an adjunct to the treatment of periodontitis in smokers: randomised clinical trial. *Benef Microbes*. 2019;10(4):375-384. https://doi.org/10.3920/bm2018.0150

36. Tyagi P, Dodwad V, Kukreja B, Kukreja P. A comparison of the efficacy of scaling and root planning with application of pomegranate chip, pomegranate gel, and scaling and root planing in sufferers with adult periodontitis - A prospective study. *Journal of Indian Society of Periodontology*. 2021;25(1):41-46. https://doi.org/10.4103/jisp.jisp_243_20

37. Tyagi P, Dodwad VW, Vaish S, Chowdhery T, Gupta N, Kukreja JB. Clinical Efficacy of Subgingivally Delivered Punica Granatum Chip and Gel in Management of Chronic Periodontitis Patients. *Kathmandu University medical journal (KUMJ)*. 2020;18(71):279-283.

38. Verma K, Dhruvakumar D, Pande M. A clinical and microbiological study to assess the efficacy of Acmella oleracea and Acacia catechu herbs as local drug delivery in treatment of chronic generalized periodontitis patients. *Journal of Indian Society of Periodontology*. 2022;26(3):254-261. https://doi.org/10.4103/jisp.jisp_264_21

39. Yaghini J, Shahabooei M, Aslani A, Zadeh MR, Kiani S, Naghsh N. Efficacy of a local-drug delivery gel containing extracts of Quercus brantii and Coriandrum sativum as an adjunct to scaling and root planing in moderate chronic periodontitis patients. *Journal of Research in Pharmacy Practice*. 2014;3(2):67-71. https://doi.org/10.4103/2279-042X.137076

40. Javid AZ, Hormoznejad R, Yousefimanesh HA, Haghighi-Zadeh MH, Zakerkish M. Impact of resveratrol supplementation on inflammatory, antioxidant, and periodontal markers in type 2 diabetic patients with chronic periodontitis. *Diabetes & metabolic syndrome*. 2019;13(4):2769‐2774. https://doi.org/10.1016/j.dsx.2019.07.042

41. Javid AZ, Maghsoumi-Norouzabad L, Ashrafzadeh E, et al. Impact of Cranberry Juice Enriched with Omega-3 Fatty Acids Adjunct with Nonsurgical Periodontal Treatment on Metabolic Control and Periodontal Status in Type 2 Patients with Diabetes with Periodontal Disease. *Journal of the American College of Nutrition*. 2018;37(1):71-79. https://doi.org/10.1080/07315724.2017.1357509

42. Jockel-Schneider Y, Stoelzel P, Hess J, Haubitz I, Fickl S, Schlagenhauf U. Impact of a Specific Collagen Peptide Food Supplement on Periodontal Inflammation in Aftercare Patients—A Randomised Controlled Trial. *Nutrients*. 2022;14(21):4473. https://doi.org/10.3390/nu14214473

43. Kaipa VRK, Asif SM, Assiri KI, et al. Antioxidant effect of spirulina in chronic periodontitis. *Medicine (United States)*. 2022;101(50):E31521. https://doi.org/10.1097/MD.0000000000031521

44. Mahendra J, Mahendra L, Muthu J, John L, Romanos GE. Clinical effects of subgingivally delivered spirulina gel in chronic periodontitis cases: A placebo controlled clinical trial. *Journal of Clinical and Diagnostic Research*. 2013;7(10):2330-2333. https://doi.org/10.7860/JCDR/2013/5793.3517

45. Manthena S, Ramoji Rao MV, Penubolu LP, Putcha M, Sri Harsha AVN. Effectiveness of coq10 oral supplements as an adjunct to scaling and root planing in improving periodontal health. *Journal of Clinical and Diagnostic Research*. 2015;9(8):ZC26-ZC28. https://doi.org/10.7860/JCDR/2015/13486.6291

46. Raut CP, Sethi KS. Comparative evaluation of co-enzyme Q10 and Melaleuca alternifolia as antioxidant gels in treatment of chronic periodontitis: A clinical study. *Contemporary clinical dentistry*. 2016;7(3):377-81. https://doi.org/10.4103/0976-237x.188572

47. Seydanur Dengizek E, Serkan D, Abubekir E, Aysun Bay K, Onder O, Arife C. Evaluating clinical and laboratory effects of ozone in non-surgical periodontal treatment: a randomized controlled trial. *Journal of applied oral science : revista FOB*. 2019;27:e20180108. https://doi.org/10.1590/1678-7757-2018-0108

48. Sravya MVN, Koduganti RR, Panthula VR, et al. Efficacy of an herbal antioxidant as an adjunct to nonsurgical periodontal therapy on procalcitonin levels in smokers with chronic periodontitis. *Journal of Indian Society of Periodontology*. 2019;23(5):430‐435. https://doi.org/10.4103/jisp.jisp_742_18

49. Stańdo M, Piatek P, Namiecinska M, Lewkowicz P, Lewkowicz N. Omega-3 Polyunsaturated Fatty Acids EPA and DHA as an Adjunct to Non-Surgical Treatment of Periodontitis: a Randomized Clinical Trial. *Nutrients*. 2020;12(9):2614. https://doi.org/10.3390/nu12092614

50. Stando-Retecka M, Piatek P, Namiecinska M, Bonikowski R, Lewkowicz P, Lewkowicz N. Clinical and microbiological outcomes of subgingival instrumentation supplemented with high-dose omega-3 polyunsaturated fatty acids in periodontal treatment - a randomized clinical trial. *BMC oral health*. 2023;23(1):290. https://doi.org/10.1186/s12903-023-03018-7

51. Surapaneni K, Koduganti RR, Ganapathi SN, et al. Efficacy of systemic administration of alpha lipoic acid and scaling and root planning in patients with chronic periodontitis and type 2 diabetes mellitus-A randomised controlle`d trial. *Journal of clinical and diagnostic research*. 2018;12(4):ZC01‐ZC05. https://doi.org/10.7860/JCDR/2018/29200.11397

52. Tawfik MS, Abdel-Ghaffar KA, Gamal AY, El-Demerdash FH, Gad HA. Lycopene solid lipid microparticles with enhanced effect on gingival crevicular fluid protein carbonyl as a biomarker of oxidative stress in patients with chronic periodontitis. *Journal of Liposome Research*. 2019;29(4):375-382. https://doi.org/10.1080/08982104.2019.1566243

53. Umrania VV, Rao Deepika PC, Kulkarni M. Evaluation of dietary supplementation of omega-3 polyunsaturated fatty acids as an adjunct to scaling and root planing on salivary interleukin-1beta levels in patients with chronic periodontitis: A clinico-immunological study. *Journal of Indian Society of Periodontology*. 2017;21(5):386-390. https://doi.org/10.4103/jisp.jisp_16_16

54. Bazyar H, Maghsoumi-Norouzabad L, Yarahmadi M, et al. The impacts of synbiotic supplementation on periodontal indices and biomarkers of oxidative stress in type 2 diabetes mellitus patients with chronic periodontitis under non-surgical periodontal therapy. A double-blind, placebo-controlled trial. *Diabetes, metabolic syndrome and obesity*. 2020;13:19‐29. https://doi.org/10.2147/DMSO.S230060

55. Butera A, Pascadopoli M, Gallo S, et al. Domiciliary Management of Periodontal Indexes and Glycosylated Hemoglobin (HbA1c) in Type 1 Diabetic Patients with Paraprobiotic-Based Toothpaste and Mousse: Randomized Clinical Trial. *Applied Sciences-Basel*. 2022;12(17):2076-3417. https://doi.org/10.3390/app12178610

56. Grusovin MG, Bossini S, Calza S, et al. Clinical efficacy of Lactobacillus reuteri-containing lozenges in the supportive therapy of generalized periodontitis stage III and IV, grade C: 1-year results of a double-blind randomized placebo-controlled pilot study. *Clin Oral Investig*. 2020;24(6):2015-2024. https://doi.org/10.1007/s00784-019-03065-x

57. Iwasaki K, Maeda K, Hidaka K, Nemoto K, Hirose Y, Deguchi S. Daily Intake of Heat-killed Lactobacillus plantarum L-137 Decreases the Probing Depth in Patients Undergoing Supportive Periodontal Therapy. *Oral Health Prev Dent*. 2016;14(3):207-14. https://doi.org/10.3290/j.ohpd.a36099

58. Laleman I, Yilmaz E, Ozcelik O, et al. The effect of a streptococci containing probiotic in periodontal therapy: a randomized controlled trial. *J Clin Periodontol*. 2015;42(11):1032-41. https://doi.org/10.1111/jcpe.12464

59. Morales A, Carvajal P, Silva N, et al. Clinical Effects of Lactobacillus rhamnosus in Non-Surgical Treatment of Chronic Periodontitis: A Randomized Placebo-Controlled Trial With 1-Year Follow-Up. *J Periodontol*. 2016;87(8):944-52. https://doi.org/10.1902/jop.2016.150665

60. Morales A, Contador R, Bravo J, et al. Clinical effects of probiotic or azithromycin as an adjunct to scaling and root planning in the treatment of stage III periodontitis: a pilot randomized controlled clinical trial. *BMC Oral Health*. 2021;21(1):12. https://doi.org/10.1186/s12903-020-01276-3

61. Poulose M, Gujar D, Panicker S, Rokade S, Guruprasad M, Gopalakrishnan D. Efficacy and Viability of Subgingival Application of Probiotics as an Adjunct to Scaling and Root Planing in Periodontitis. *Indian J Dent Res*. 2024;35(1):59-64. https://doi.org/10.4103/ijdr.ijdr_533_23

62. Ramos TCS, Boas MLV, Nunes CMM, et al. Effect of systemic antibiotic and probiotic therapies as adjuvant treatments of subgingival instrumentation for periodontitis: a randomized controlled clinical study. *J Appl Oral Sci*. 2022;30:e20210583. https://doi.org/10.1590/1678-7757-2021-0583

63. Soares LG, Carvalho EB, Tinoco EMB. Clinical effect of Lactobacillus on the treatment of severe periodontitis and halitosis: A double-blinded, placebo-controlled, randomized clinical trial. *Am J Dent*. 2019;32(1):9-13.

64. Jayachandran P, Menon KS, Kurup S, et al. Influence of Vitamin D & calcium supplementation in the management of periodontitis. *Journal of Clinical and Diagnostic Research*. 2015;9(6):ZC35-ZC38. https://doi.org/10.7860/JCDR/2015/12292.6091

65. Nagate RR, Yuvaraja M, AlQahtani SM, et al. Efficacy of Pluronic F-127 gel containing green tea catechin extract on chronic periodontitis - A clinical study. *Tropical Journal of Pharmaceutical Research*. 2020;19(2):427-432. https://doi.org/10.4314/tjpr.v19i2.27

66. Gao W, Tang H, Wang D, Zhou X, Song Y, Wang Z. Effect of short-term vitamin D supplementation after nonsurgical periodontal treatment: a randomized, double-masked, placebo-controlled clinical trial. *Journal of periodontal research*. 2020;55(3):354‐362. https://doi.org/10.1111/jre.12719

67. Kalsi R, Bhushan KS, Mathur MK, Gupta V, Gupta DK, Pandey SK. To Evaluate the Effect of Vitamin B Complex on Wound Healing – A Clinical and Microbiological Study. *Journal of Pharmacy and Bioallied Sciences*. 2024;16:S549-S551. https://doi.org/10.4103/jpbs.jpbs_856_23

68. Neiva RF, Al-Shammari K, Nociti FH, Soehren S, Wang HL. Effects of vitamin-B complex supplementation on periodontal wound healing. *Journal of periodontology*. 2005;76(7):1084‐1091. https://doi.org/10.1902/jop.2005.76.7.1084

69. Ramaprabha G, Khan NS, Kunusoth R, Kakati I, Hussain Qadri SS, Seshadri PR. Assessment of outcome of oral supplementation of Vitamin D3 as an adjunct to scaling and root planing in chronic periodontitis patients with type II diabetes mellitus - A randomized controlled clinical trial. *Journal of pharmacy & bioallied sciences*. 2023;15(5):S346‐S349. https://doi.org/10.4103/jpbs.jpbs_543_22

70. Sulaiman AEA, Shehadeh RMH. Assessment of total antioxidant capacity and the use of vitamin C in the treatment of non-smokers with chronic periodontitis. *Journal of Periodontology*. 2010;81(11):1547-1554. https://doi.org/10.1902/jop.2010.100173

71. Isola G, Polizzi A, Iorio-Siciliano V, Alibrandi A, Ramaglia L, Leonardi R. Effectiveness of a nutraceutical agent in the non-surgical periodontal therapy: a randomized, controlled clinical trial. *Clinical oral investigations*. 2021;25(3):1035‐1045. https://doi.org/10.1007/s00784-020-03397-z

72. Laky B, Bruckmann C, Blumenschein J, Durstberger G, Haririan H. Effect of a multinutrient supplement as an adjunct to nonsurgical treatment of periodontitis: a randomized placebo-controlled clinical trial. *Journal of periodontology*. 2024;95(2):101‐113. https://doi.org/10.1002/JPER.23-0115

73. Lee J, Park JC, Jung UW, et al. Improvement in periodontal healing after periodontal surgery supported by nutritional supplement drinks. *Journal of periodontal & implant science*. 2014;44(3):109‐117. https://doi.org/10.5051/jpis.2014.44.3.109

74. Anton DM, Martu MA, Maris M, et al. Study on the Effects of Melatonin on Glycemic Control and Periodontal Parameters in Patients with Type II Diabetes Mellitus and Periodontal Disease. *Medicina (Kaunas, Lithuania)*. 2021;57(2):140. https://doi.org/10.3390/medicina57020140

75. Bazyar H, Gholinezhad H, Moradi L, et al. The effects of melatonin supplementation in adjunct with non-surgical periodontal therapy on periodontal status, serum melatonin and inflammatory markers in type 2 diabetes mellitus patients with chronic periodontitis: a double-blind, placebo-controlled trial. *Inflammopharmacology*. 2019;27(1):67‐76. https://doi.org/10.1007/s10787-018-0539-0

76. El‐Sharkawy H, Elmeadawy S, Elshinnawi U, Anees M. Is dietary melatonin supplementation a viable adjunctive therapy for chronic periodontitis?—A randomized controlled clinical trial. *Journal of periodontal research*. 2019;54(2):190‐197. https://doi.org/10.1111/jre.12619

77. Gonde NP, Rathod SR, Kolte AP. Comparative evaluation of 1% melatonin gel in the treatment of intrabony defect: a randomized controlled clinical trial. *Journal of periodontology*. 2022;93(12):1878‐1888. https://doi.org/10.1002/JPER.21-0515

78. Marawar AP, Marawar PP, Nandal DH, Tilak AV, Bhalsinge RB, Barde AA. Therapeutic potential of melatonin in periodontitis: a randomised, placebo controlled, double blind study. *Research journal of pharmaceutical, biological and chemical sciences*. 2014;5(4):31‐39.

79. Montero J, Lopez-Valverde N, Ferrera M-J, Lopez-Valverde A. Changes in crevicular cytokines after application of melatonin in patients with periodontal disease. *Journal of clinical and experimental dentistry*. 2017;9(9):e1081-e1087. https://doi.org/10.4317/jced.53934


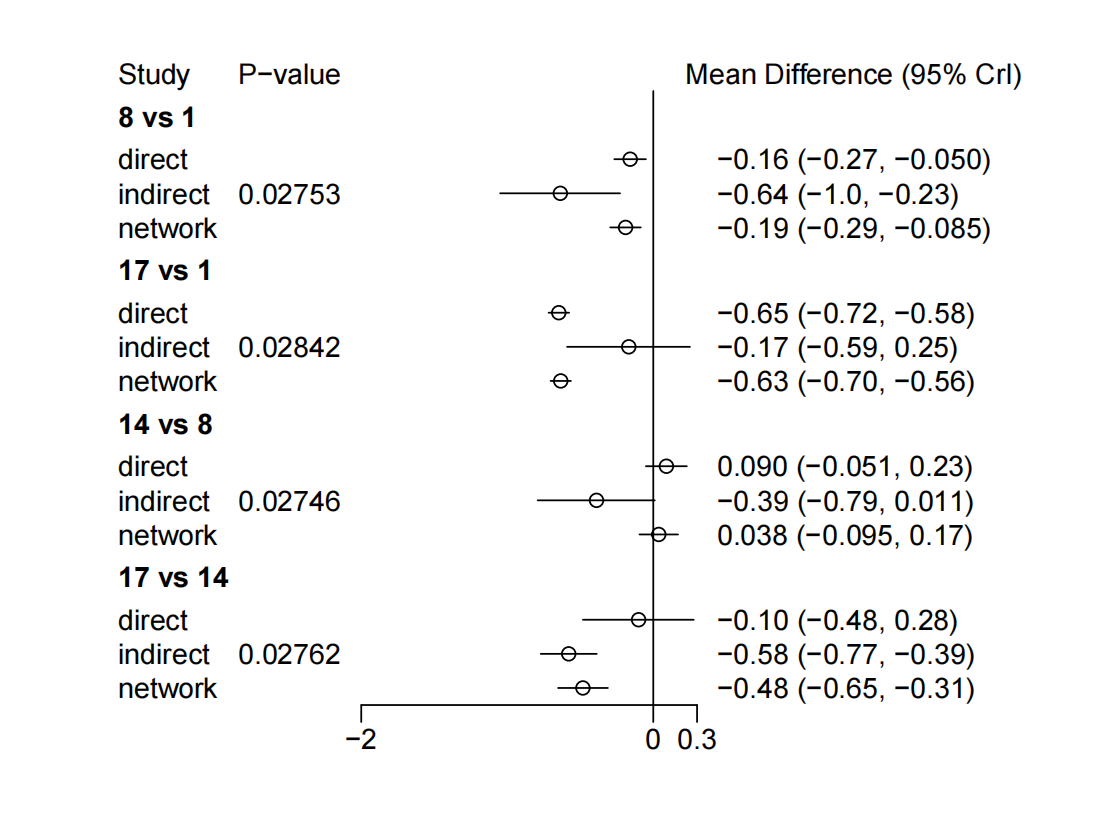


**Figure S1** The result of the inconsistency test in GI.


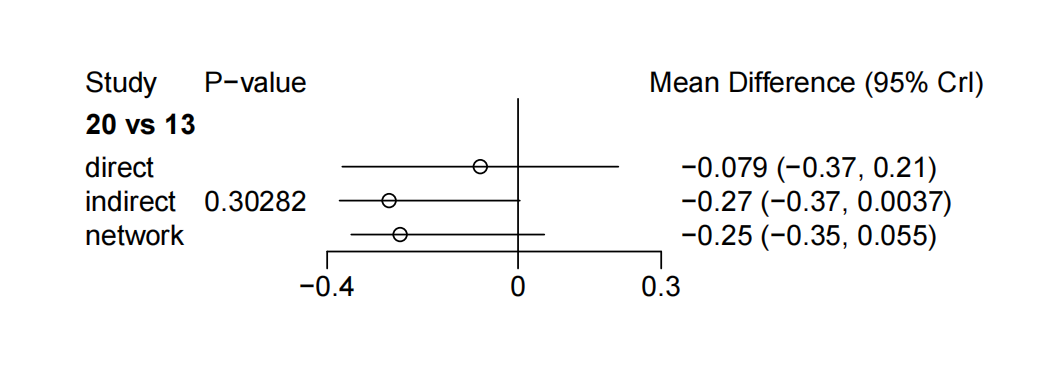


**Figure S2** The result of the inconsistency test in PI.


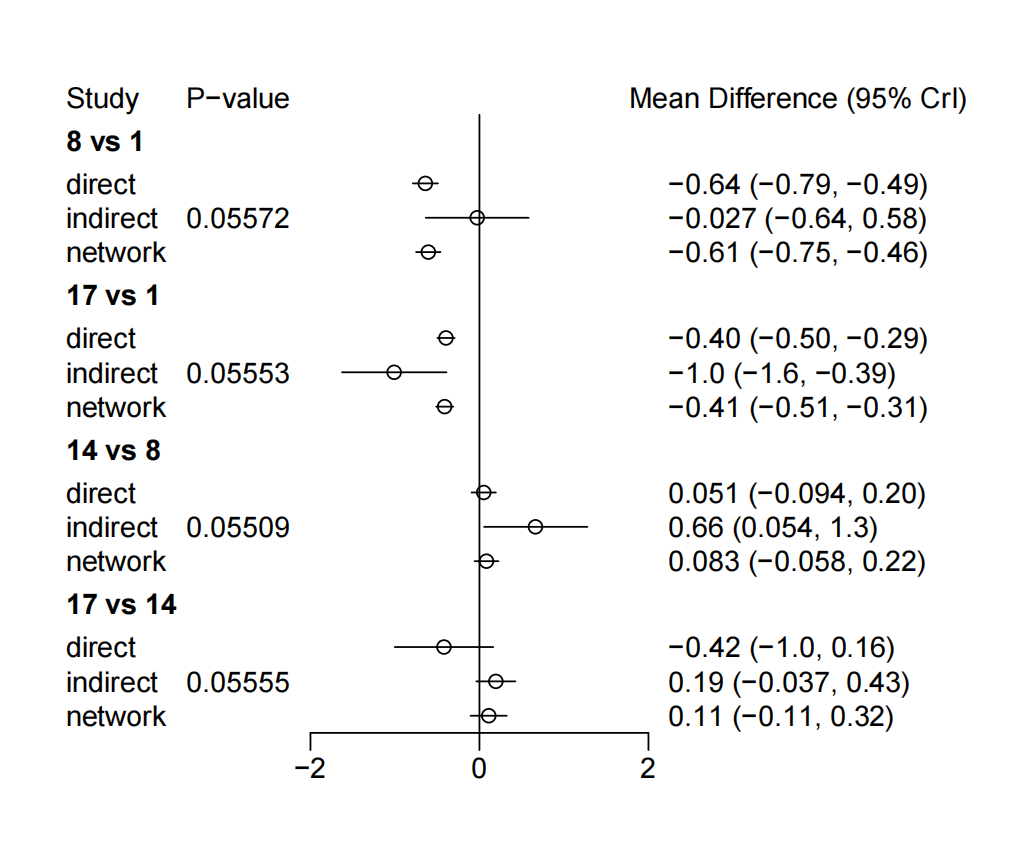


**Figure S3** The result of the inconsistency test in PPD.


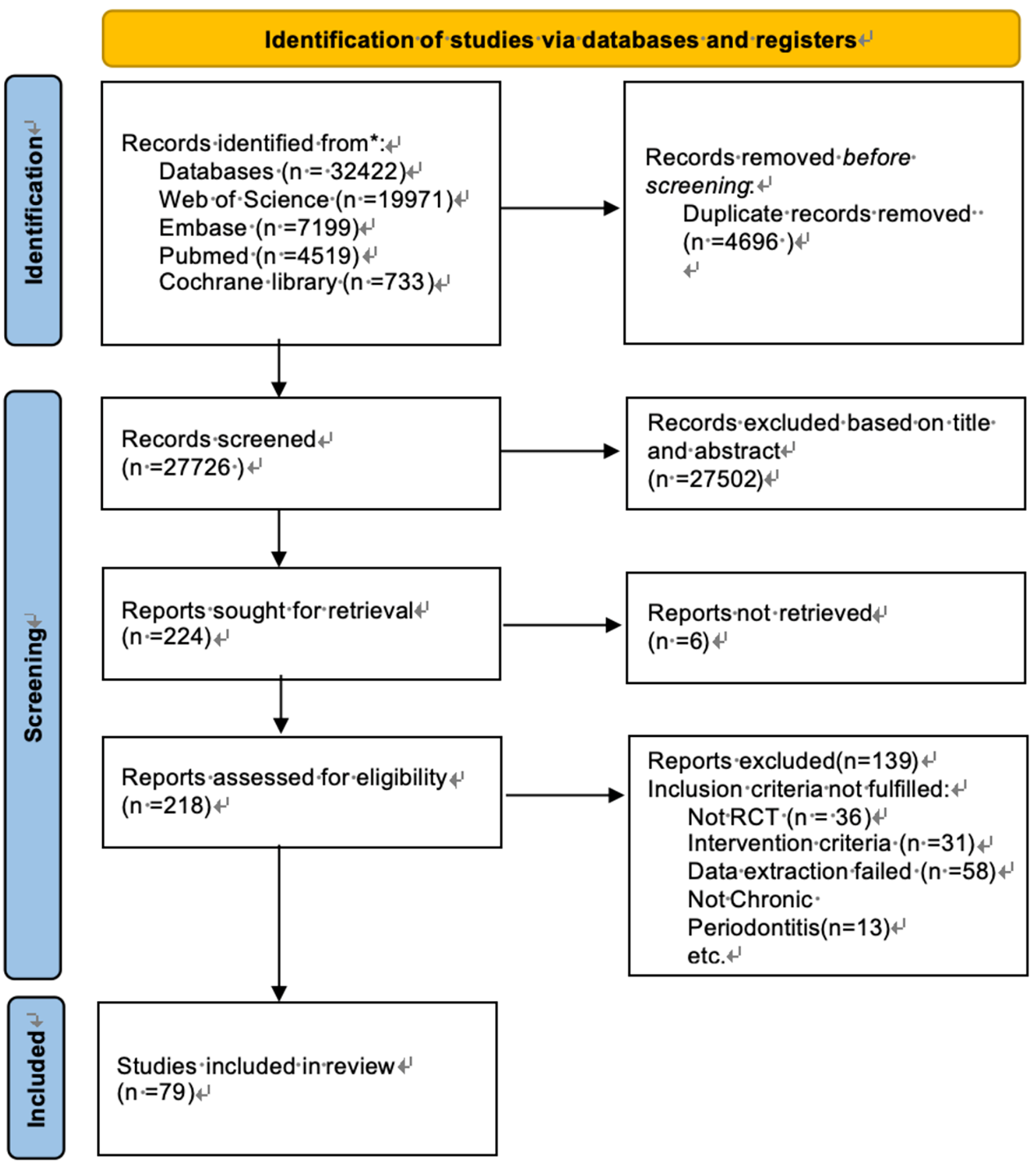


**Figure S4** Prisma® flow diagram demonstrating the search and selection strategy and results.


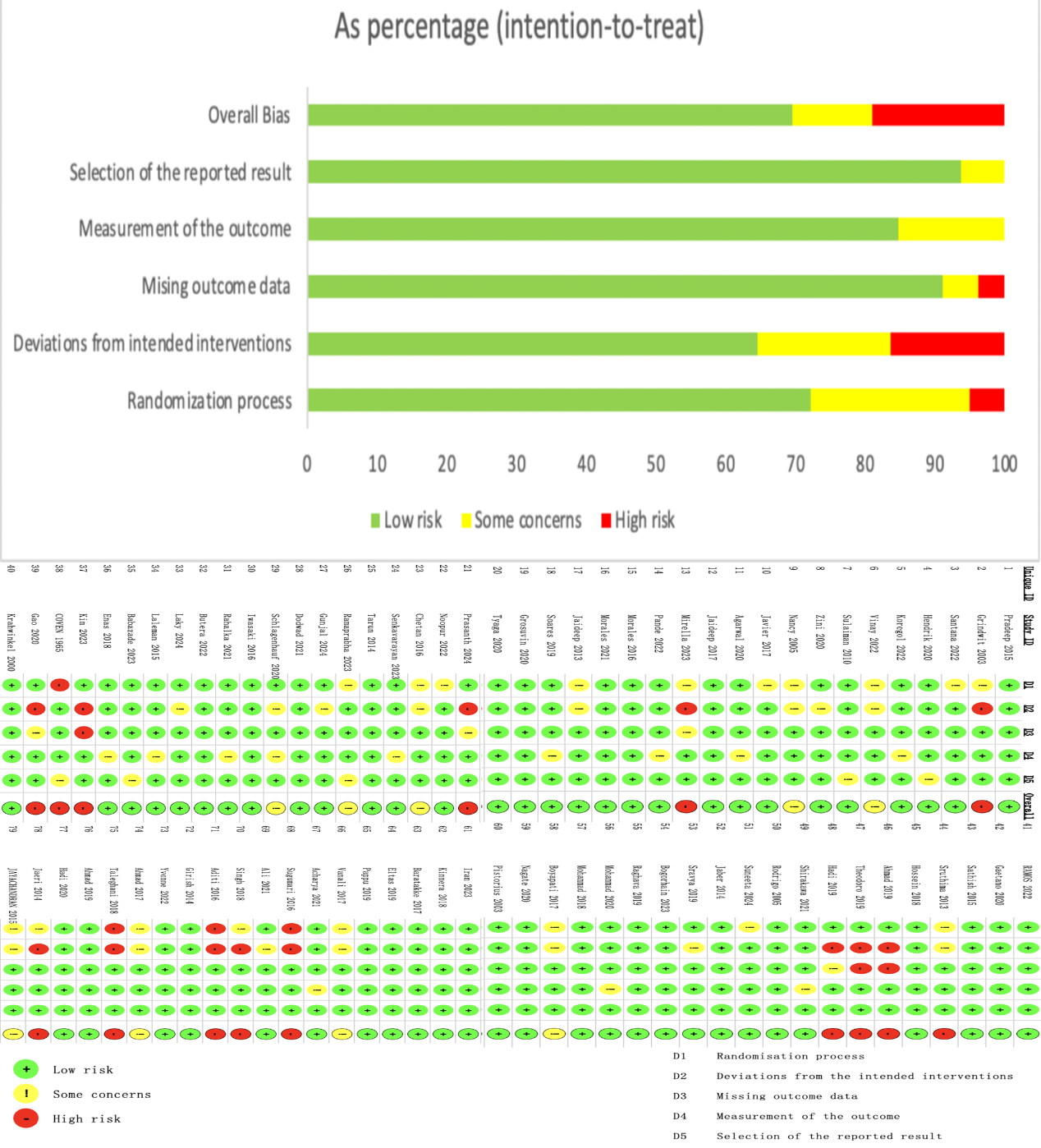


**Figure S5** RoB assessment result.


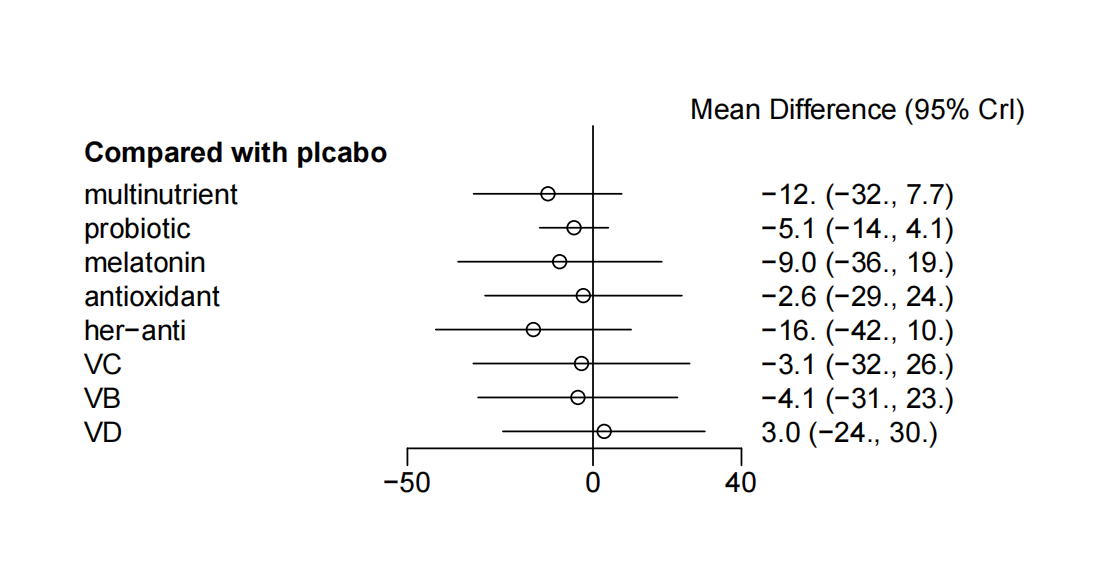


**Figure S6** Forest plot for the decline in BOP in different intervention.


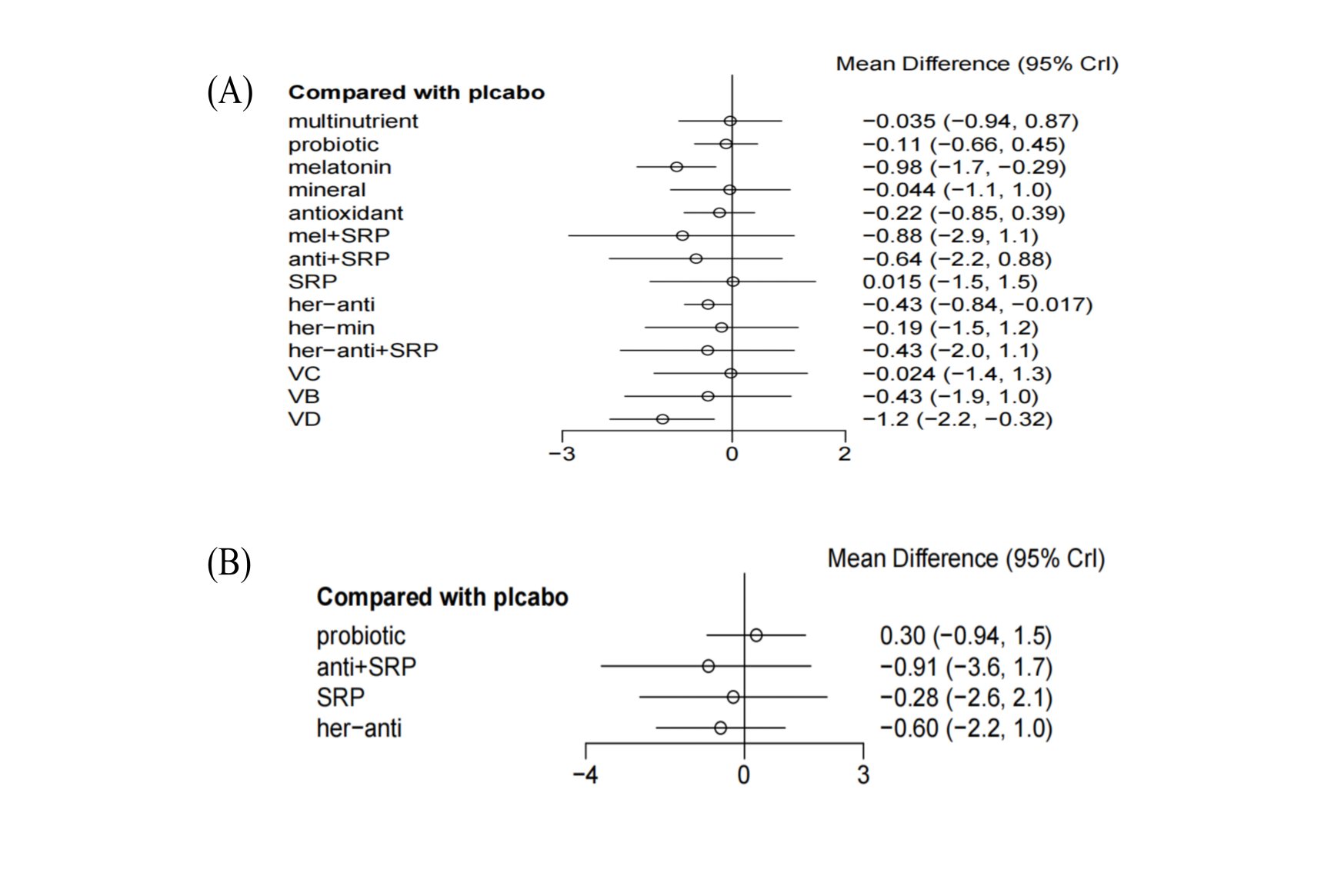


**Figure S7** Forest plot for the decline in CAL in different intervention. A-3m; B-6m.


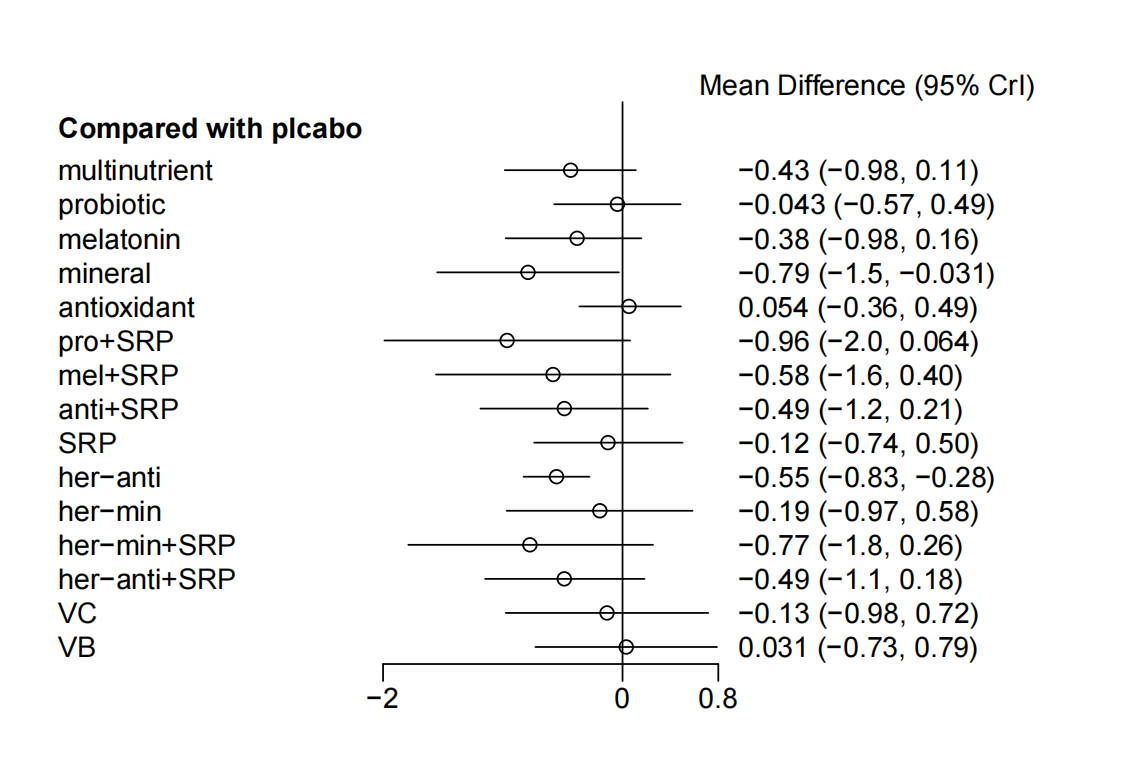


**Figure S8** Forest plot for the decline in GI in different intervention.


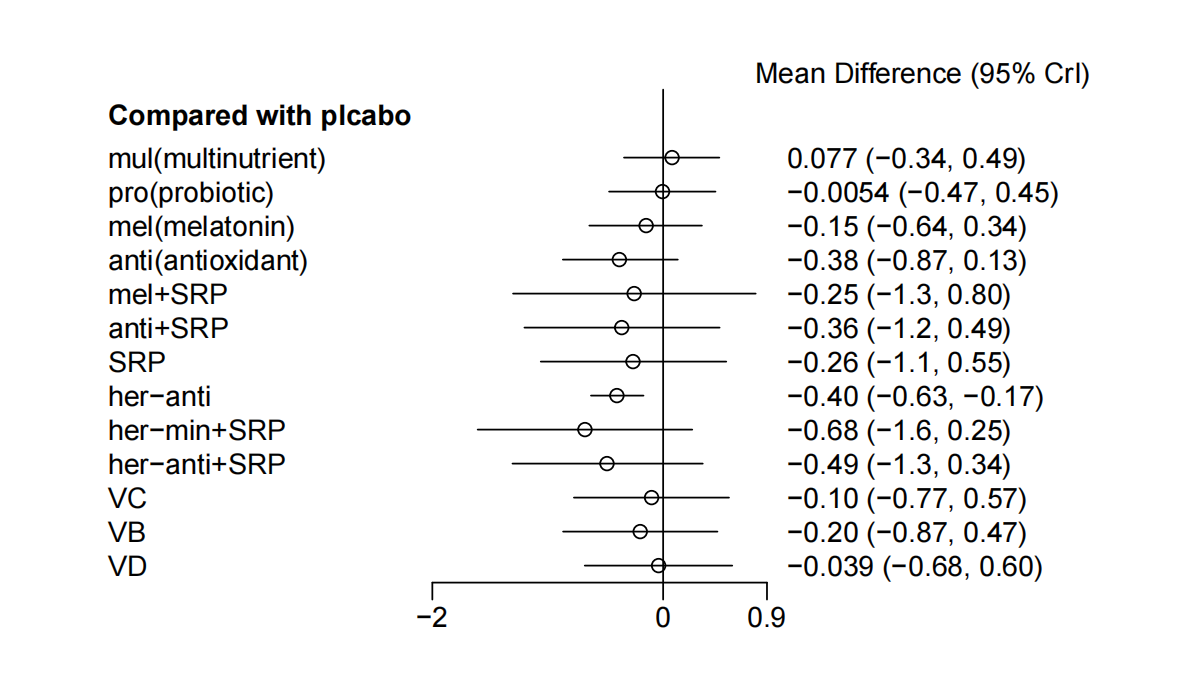


**Figure S9** Forest plot for the decline in PI in different interventions.


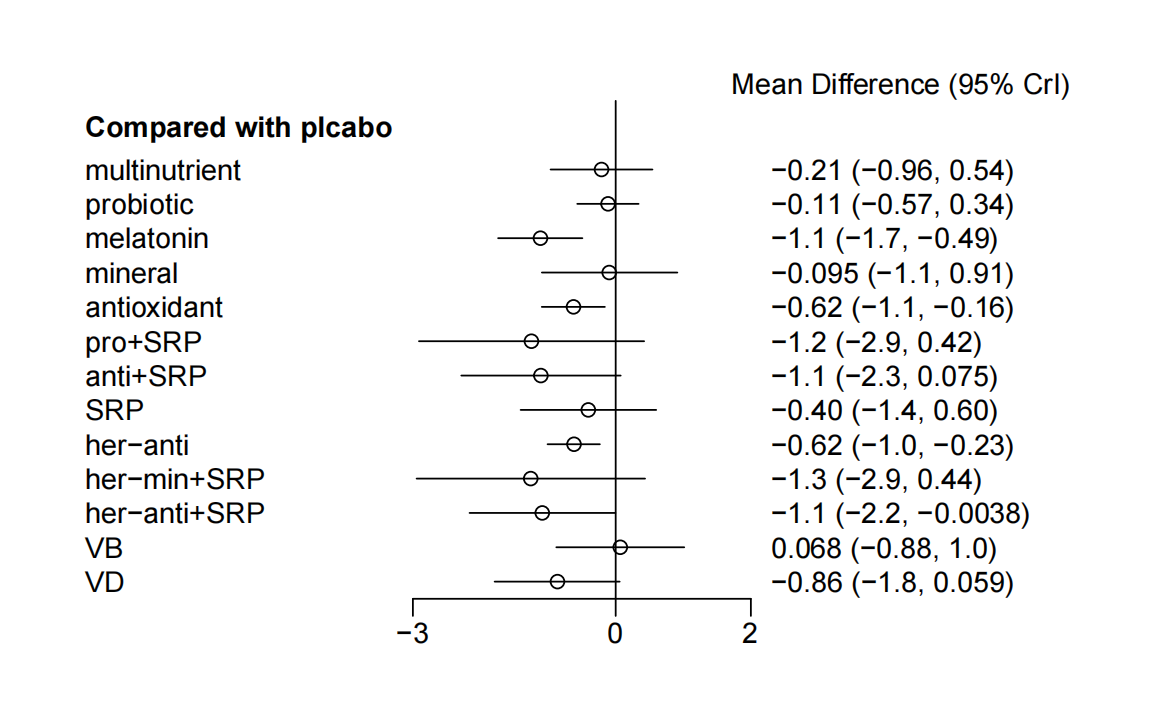


**Figure S10** Forest plot for the decline in PPD in different interventions.


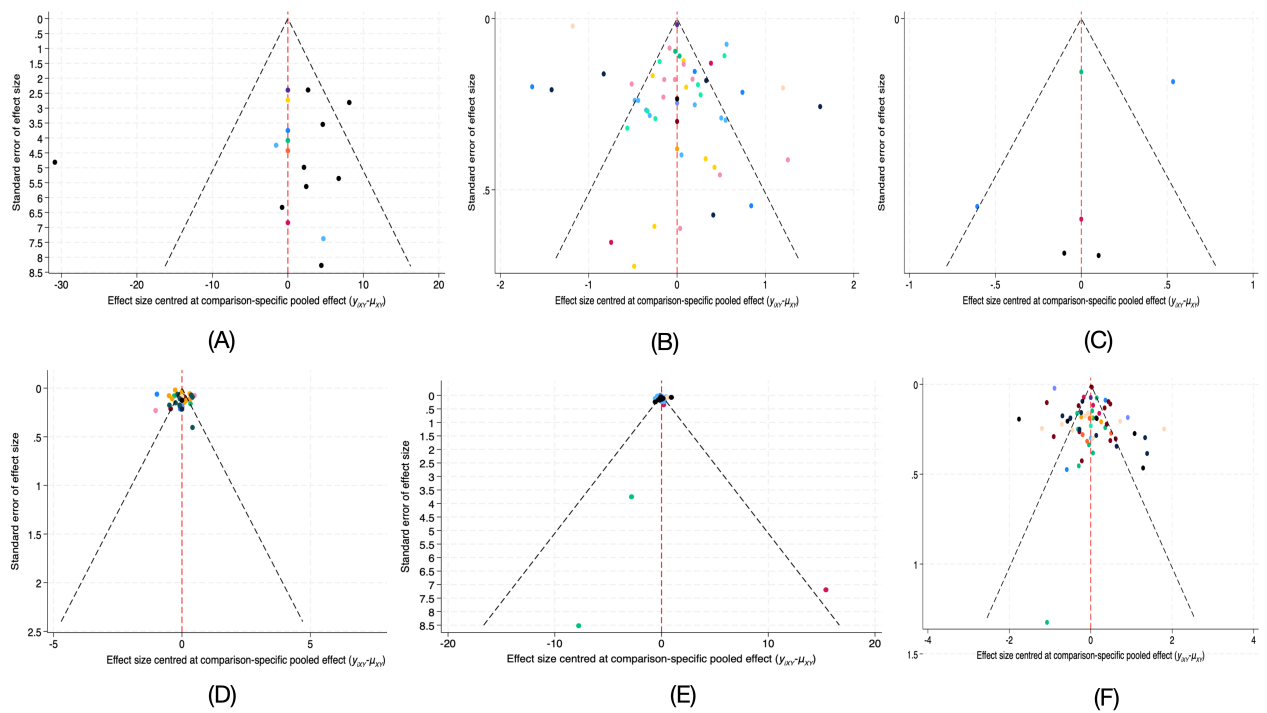


**Figure S11** Funnel plots for different outcomes: (a) BOP; (b) CAL(3m); (c) CAL(6m); (d) GI; (e) PI; (f) PPD.

**Table S1** Specific search strategy for PubMed.

| Search number | Query | Results |
| --- | --- | --- |
| 1 | Dietary Supplements[MeSH Terms] | 106,962 |
| 2 | "diet additive"[Title/Abstract] OR "diet supplement"[Title/Abstract] OR "Dietary Supplement*"[Title/Abstract] OR "food supplement*"[Title/Abstract] OR "Herbal Supplement*"[Title/Abstract] OR "Neutraceutical*"[Title/Abstract] OR "Nutraceutical*"[Title/Abstract] OR "Nutriceutical*"[Title/Abstract] OR "nutritional supplement"[Title/Abstract] OR "supplementary diet"[Title/Abstract] | 50,714 |
| 3 | Melatonin[MeSH Terms] | 24,131 |
| 4 | Antioxidants[MeSH Terms] | 178,029 |
| 6 | "Anti Oxidant Effect*"[Title/Abstract] OR "Anti Oxidant*"[Title/Abstract] OR "Antioxidant Activity"[Title/Abstract] OR "antioxidant agent"[Title/Abstract] OR "Antioxidant Effect*"[Title/Abstract] OR "antioxidant nutrient"[Title/Abstract] OR "Antioxidant*"[Title/Abstract] OR "antioxidative"[Title/Abstract] OR "Endogenous Antioxidant"[Title/Abstract] OR "Endogenous Antioxidants"[Title/Abstract] OR "phenolic antioxidant "[Title/Abstract] | 334,840 |
| 7 | Vitamins[MeSH Terms] | 46,075 |
| 8 | "davitamin"[Title/Abstract] OR "vitamin*"[Title/Abstract] OR "vitaminology"[Title/Abstract] | 267,055 |
| 9 | Plant Extracts[MeSH Terms] | 217,273 |
| 10 | "Herbal Medicines"[Title/Abstract] OR "Plant Extract*"[Title/Abstract] | 23,797 |
| 11 | Periodontal Diseases[MeSH Terms] | 98,391 |
| 12 | "dental loss"[Title/Abstract] OR "dental migration"[Title/Abstract] OR "dental mobility"[Title/Abstract] OR "furcation defects"[Title/Abstract] OR "mesial movement of teeth"[Title/Abstract] OR "paradontal disease"[Title/Abstract] OR "paradontopathy"[Title/Abstract] OR "parodontal disease"[Title/Abstract] OR "Parodontos*"[Title/Abstract] OR "peridontal disease"[Title/Abstract] OR "periodontal atrophy"[Title/Abstract] OR "periodontal attachment loss"[Title/Abstract] OR "periodontal disease*"[Title/Abstract] OR "periodontal infection"[Title/Abstract] OR "periodontium disease"[Title/Abstract] OR "periodontopathy"[Title/Abstract] OR "Pyorrhea Alveolaris"[Title/Abstract] OR "tooth loss"[Title/Abstract] OR "tooth migration"[Title/Abstract] OR "tooth mobility"[Title/Abstract] OR "tooth movement''[Title/Abstract] | 41,257 |
| 13 | minerals[MeSH Terms] | 195,142 |
| 14 | (#1 OR #2 OR #3 OR #4 OR #6 OR #7 OR #8 OR #9 OR #10 OR #13) AND (#12 OR #11) | 4,519 |

**Table S2** DIC of different periodontal parameters.

|  | consistency | ume |
| --- | --- | --- |
| PI | 189.00053 | 188.93201 |
| BOP | 66.63778 | 66.71799 |
| GI | 183.11750 | 182.83628 |
| CAL(3m) | 215.9338 | 215.9444 |
| CAL(6m) | 23.87355 | 23.89523 |
| PPD | 253.8001 | 253.9877 |

**Table S3** Overview of included studies in this NMA.

|  | First Author | Publication years | Study Design | region | Periodontitis Type(Grade) | extent diagnosis | No. of subjects | | gender(male/female) | | age | | Intervention | | evaluation period | Outcomes |
| --- | --- | --- | --- | --- | --- | --- | --- | --- | --- | --- | --- | --- | --- | --- | --- | --- |
|  |  |  |  |  |  |  | experimental | control | E | C | E | C | E | C |  |  |
| Adjunctive Local Delivery of Aloe Vera Gel in Type 2 Diabetics With  Chronic Periodontitis : A Randomized Controlled Clinical Trial | A R Pradeep | 2016 | RCT | Bengaluru | chronic periodontitis(II) | type 2 diabetes mellitus | 30 | 30 | 17/13 | 16/14 | 35.03 ± 4.78 | 34.76 ± 5.57 | Adjunctive Local Delivery of Aloe Vera Gel | Placebo | 3m,6m | plaque index(PI), probing depth (PD) ,clinical attachment level (CAL) |
| Anti-inflammatory potential of an essential oil-containing mouthwash in elderly subjects enrolled in supportive periodontal therapy: a 6-week randomised controlled clinical trial | Hendrik Jünger | 2020 | RCT | Switzerland | chronic periodontitis(I-III) | Blank | 24 | 24 | 13/11 | 12/12 | 78.7 ± 7.9 | 76.2 ± 6.5 | sage-containing mouthwash | placebo | 6w | plaque index(PI) |
| Antioxidant effect of spirulina in chronic  periodontitis | Vinay Rama Krishna Kaipa | 2022 | RCT | India | chronic periodontitis(II-III) | Blank | 30 | 30 | Not metioned | Not metioned | 35-65 | Not metioned | Spirulina microspheres+SRP | SRP+placebo | 90d | bleeding on probing(BOP), Clinical attachment Level(CAL), Gingival index(GI) and probing pocket depth (PPD) |
| Assessment of Total Antioxidant Capacity and the Use of Vitamin C in the Treatment of Non-Smokers With Chronic Periodontitis | Ali E. Abou Sulaiman | 2010 | RCT | Syria | chronic periodontitis(I-II) | Blank | 15 | 15 | 12/3 | 9/6 | 23-65 | 30-60 | dose of vitamin C | placebo | 3m | probing depth(PD),Clinical attachment level(CAL),bleeding on probing(BOP),plaque index(PI),Gingival index(GI) |
| Changes in crevicular cytokines after application of melatonin  in patients with periodontal disease | Javier Montero | 2017 | RCT | Spain | chronic periodontitis(I-III) | diabetes mellitus | 30 | 30 | 14/16 | 13/17 | 43.1±12.4 | 45.46±8.8 | melatonin(1% orabase cream  formula) | placebo | 20d | Gingival index(GI),Pocket depth（PD） |
| Clinical and microbiological effects of 1% Matricaria chamomilla mouth rinse on chronic periodontitis: A double-blind randomized placebo controlled trial | Agarwal, A. | 2020 | RCT | India | chronic periodontitis(II-III) | Blank | 25 | 25 | 12/13/12/13 | 15/10 | 51.43±9.88/49.12±9.77 | 54.76±8.13 | SRP + 1% MTC mouth rinse | SRP + placebo | 3m | plaque index(PI), Gingival index(GI), probing pocket depth [PPD], Clinical attachment level(CAL） |
| Clinical and microbiological efficacy of 4% Garcinia mangostana L. pericarp gel as local drug delivery in the treatment of chronic  periodontitis: A randomized, controlled clinical trial | Jaideep Mahendra | 2017 | RCT | India | chronic periodontitis(II-III) | Blank | 25 | 25 | 27/23 | Not metioned | Not metioned | Not metioned | SRP and the subgingival application of mangostana gel | SRP+placebo | 3m | probing pocket depth（PPD）, Clinical attachment level(CAL), plaque index(PI) |
| Clinical and microbiological outcomes  of subgingival instrumentation supplemented  with high‑dose omega‑3 polyunsaturated fatty  acids in periodontal treatment – a randomized  clinical trial | Mirella Stańdo‑Retecka | 2023 | RCT | Poland | chronic periodontitis(III) | Blank | 20 | 20 | 8/12 | 11/9 | 44.1±7.7 | 52.2±10.8 | SRP plus omega-3 PUFAs | SRP | 3m | probing depth(PD), CAL Clinical attachment level(CAL), bleeding on probing(BOP),plaque index(PI) |
| A clinical and microbiological study to  assess the efficacy of Acmella oleracea and Acacia catechu herbs as local  drug delivery in treatment of chronic  generalized periodontitis patients | Milind Pande | 2022 | RCT | India | chronic periodontitis(II-III) | Blank | 10 | 10 | Not metioned | Not metioned | 25-65 | Not metioned | A. oleracea (1%)/ A. catechu (1%) as local drug delivery | SRP | 3m,6m | Gingival index(GI), plaque index(PI), pocket probing depth(PPD), Clinical attachment level(CAL) |
| Clinical Effects of Lactobacillus Rhamnosus in Non-Surgical  Treatment of Chronic Periodontitis: A Randomized Placebo_x005f_x005f_x0002_Controlled Trial With 1-Year Follow-up | Alicia Morales | 2016 | RCT | Chile | chronic periodontitis(III) | Blank | 14 | 14 | 7/7 | 7/7 | 52.7 ± 7.3 | 46.9 ± 10.3 | SRP + probiotic | SRP + placebo | 3m,6m | bleeding on probing(BOP), pocket depths (PD),clinical attachment level(CAL） |
| Clinical effects of probiotic or azithromycin as an adjunct to scaling and root planning in the treatment of stage III periodontitis: a pilot randomized controlled clinical trial | Morales, A. | 2021 | RCT | Chile | chronic periodontitis(III) | Blank | 16/16（2groups） | 15 | 8/8/10/6 | 8/7 | 46.5±9.3/49.0±7.9 | 52.8±7.5 | probiotics | placebo | 3m,6m | Probing pocket depth (PPD), bleeding on probing(BOP), clinical attachment loss (CAL),plaque accumulation (PI) |
| Clinical Effects of Subgingivally Delivered  Spirulina Gel in Chronic Periodontitis Cases:  A Placebo Controlled Clinical Trial | Jaideep Mahendra | 2013 | RCT | India | chronic periodontitis(II-III) | Blank | 33（sites） | 31（sites） | Not metioned | Not metioned | 25-45 | Not metioned | SRP along with spirulina gel | SRP | 120d | Probing Pocket Depth (PPD),Clinical attachment loss(CAL) |
| Clinical effect of Lactobacillus on the treatment of severe periodontitis and halitosis: A double-blinded, placebo-controlled, randomized clinical trial | Soares, L. G. | 2019 | RCT | Brazil | chronic periodontitis(III) | Blank | 30 | 30 | 24/36 | Not metioned | 57.0±10.57 | Not metioned | Lactobacillus reuteri, salivarius and acidophilus | placebo | 90d | plaque index(PI),Probing Pocket Depth (PPD)，clinical attachment level (CAL)，Bleeding on probing (BOP) |
| Clinical efficacy of Lactobacillus reuteri-containing lozenges in the supportive therapy of generalized periodontitis stage III and IV, grade C: 1-year results of a double-blind randomized placebo-controlled pilot study | Maria Gabriella Grusovin | 2020 | RCT | Italy | chronic periodontitis(III) | Blank | 10 | 10 | 8/12 | Not metioned | 31-70 | Not metioned | probiotics | placebo | 3m,6m | probing pockets depth(PPD),bleeding on probing(BOP) |
| Clinical Efficacy of Subgingivally Delivered Punica Granatum  Chip and Gel in Management of Chronic Periodontitis  Patients | Tyagi, P. | 2020 | RCT | India | chronic periodontitis(I-III) | Blank | 10/10（2groups） | 10 | Not metioned | Not metioned | 35-50 | Not metioned | SRP+Punica granatum chip/SRP+ Punica granatum gel | SRP | 45d | plaque index(PI), Gingival index(GI), probing pocket depth (PPD) |
| Clinico-immunological evaluation of use of omega- 3 fatty acids as nutraceutical approach in management of patients with chronic periodontitis: A randomized clinical trial | T. Prasanth | 2024 | RCT | India | chronic periodontitis(I-III) | Blank | 30 | 30 | 37/23 | Not metioned | 18-65 | Not metioned | SRP+ a dietary supplementation of omega-3 fatty acid | SRP | 3m,6m | plaque index(PI), pocket probing depth (PPD),Clinical attachment level(CAL) |
| Comparative evaluation of 1% melatonin gel in the treatment of intrabony defect: A randomized controlled clinical trial | Noopur P. Gonde | 2022 | RCT | India | chronic periodontitis(III) | Blank | 22 | 22 | Not metioned | Not metioned | Not metioned | Not metioned | melatonin gel | placebo | 3m,6m | probing depth (PD), Clinical attachment level(CAL), plaque index(PI) |
| Comparative evaluation of co-enzyme Q10 and Melaleuca alternifolia as  antioxidant gels in treatment of chronic periodontitis: A clinical study | Chetan Purushottam Raut | 2016 | RCT | India | chronic periodontitis(II-III) | Blank | 15sites/15sites | 15sites | Not metioned | Not metioned | Not metioned | Not metioned | SRP+plusCoenzymeQ10 gel delivery/SRP plus tea tree oil （TTO） gel delivery | SRP + placebo | 1m | plaque index(PI), gingival bleeding index (GI), probing pocket depth (PPD), Clinical attachment loss(CAL) |
| Comparative evaluation of  subgingivally delivered xanthan-based  chlorhexidine gel and herbal extract  gel in the treatment of chronic  periodontitis | Tarun Rana | 2014 | RCT | India | chronic periodontitis(II-III) | Blank | 30sites/30sites/30sites/30sites | 30sites | Not metioned | Not metioned | 30-50 | Not metioned | SRP+Herbal gel/Herbal gel | SRP | 3m | plaque index(PI), Gingival index(GI), probing pocket depth （PPD）Clinical attachment level(CAL) |
| Assessment of Outcome of Oral Supplementation of Vitamin D3 as an  Adjunct to Scaling and Root Planing in Chronic Periodontitis Patients  with Type II Diabetes Mellitus – A Randomized Controlled Clinical Trial | Ramaprabha G | 2023 | RCT | India | chronic periodontitis(I-III) | type 2 diabetes mellitus | 25/25 | 21/21 | Not metioned | Not metioned | 35-60 | Not metioned | oral vitamin D3 granules | blank | 8w | plaque index(PI), Probing Pocket Depth (PPD), Clinical attachment level(CAL) |
| Comparison of the effectiveness of Morus alba and  chlorhexidine gels as an adjunct to scaling and root planing on  stage II periodontitis – A randomized controlled clinical trial | Shilpa Gunjal | 2024 | RCT | India | chronic periodontitis(II) | Blank | 60/60（2groups） | 60 | 34/26/31/29 | 30/30 | 44.78 ± 5.16/44.68 ± 5.86 | 44.91 ± 6.20 | SRP+chlorhexidine digluconate/SRP+Morus alba(MA) | SRP | 45d | plaque index(PI), Gingival index(GI), probing pocket depth (PPD) |
| A comparison of the efficacy of scaling  and root planning with application  of pomegranate chip, pomegranate  gel, and scaling and root planing in  sufferers with adult periodontitis –  A prospective study | Vidya Dodwad | 2021 | RCT | India | chronic periodontitis(II-III) | Blank | 10/10(2groups） | 10 | 12/18 | Not metioned | 42.7(35-50) | Not metioned | medicated chips/gel | placebo | 45d | plaque index(PI), Gingival index(GI), probing pocket depth（PPD） |
| Daily Intake of Heat-killed Lactobacillus plantarum L-137 Decreases the Probing Depth in Patients Undergoing Supportive Periodontal Therapy | Iwasaki, K. | 2016 | RCT | Japan | chronic periodontitis(I-III) | Blank | 19 | 17 | 6/13 | 7/10 | 68.2±9.7 | 67.0±8.1 | HK L-137 capsule | placebo | 12w | plaque index(PI), Gingival index(GI), bleeding on probing(BOP), probing depth (PD) |
| Determination of efficacy of curcumin  and Tulsi extracts as local drugs in  periodontal pocket reduction: A clinical  and microbiological study | Apurva Rahalkar | 2021 | RCT | India | chronic periodontitis(II-III) | Blank | 15sites/15sites | 15sites | 5/10 | Not metioned | 37-57 | Not metioned | LDD of  curcumin extract+SRP/SRP+LDD of Tulsi extract | SRP | 30d | Probing Pocket Depth（PPD）, Clinical attachment level(CAL), plaque index(PI), Gingival index(GI） |
| Domiciliary Management of Periodontal Indexes and Glycosylated Hemoglobin (HbA1c) in Type 1 Diabetic Patients with Paraprobiotic-Based Toothpaste and Mousse: Randomized Clinical Trial | Andrea Butera | 2022 | RCT | Italy | chronic periodontitis(I-III) | type 1 diabetes mellitus | 20 | 20 | 22/18 | Not metioned | 35.15 ± 9.03 | 34.95 ± 8.48 | home oral care protocol：probiotic-based | placebo | 3m | Probing Pocket Depth (PPD), plaque index(PI), Clinical attachment loss(CAL), bleeding on probing(BOP) |
| Effect of a multinutrient supplement as an adjunct to nonsurgical treatment of periodontitis: A randomized placebo-controlled clinical trial | Brenda Laky | 2024 | RCT | Austria | chronic periodontitis(III) | Blank | 20 | 19 | 12/8 | 11/8 | 44.0 ± 9.3 | 48.9 ± 10.8 | multinutrient supplement | placebo | 2m | probing pocket depth（PPD）, bleeding on probing(BOP), plaque index(PI) |
| The effect of a streptococci containing probiotic in periodontal therapy: a randomized controlled trial | Isabelle Laleman | 2015 | RCT | Turkey | chronic periodontitis(I-III) | Blank | 24 | 24 | 12/12 | 14/10 | 46 ±5 | 47±5 | SRP+probiotic tablet | SRP + placebo | 12w | The pocket probing depth（PPD）, bleeding on probing(BOP), plaque and gingival indices（PI） |
| Effect of Omega-3 Fatty Acids on Chronic Periodontitis  Patients in Postmenopausal Women:  A Randomised Controlled Clinical Study | Enas Ahmed Elgendy | 2018 | RCT | Egypt | chronic periodontitis(I-III) | Blank | 25 | 25 | Not metioned | Not metioned | 45-60 | Not metioned | SRP +omega-3 FAs | SRP+ SRP | 3m | plaque index(PI), Gingival index(GI), probing pocket depth (PPD), Clinical attachment level(CAL) |
| Efect of oral gargle containing Lespedeza  cuneata extract on periodontal health  improvement and disease prevention:  a randomized, controlled clinical trial | Yu‑Rin Kim | 2023 | RCT | Republic of Korea | chronic periodontitis(I-III) | caries | 32 | 31 | 6/26 | 6/25 | 27.75±8.33 | 27.94±8.40 | Lespedeza cuneata(LC) extract | placebo | 5d | plaque index(PI), and Gingival index(GI) |
| Effect of short-term vitamin D supplementation after  nonsurgical periodontal treatment: A randomized, double_x005f_x005f_x005f_x005f_x005f_x005f_x005f_x005f_x005f_x005f_x005f_x005f_x005f_x0002_masked, placebo-controlled clinical trial | Weimin Gao | 2020 | RCT | China | chronic periodontitis(II-III) | Blank | 120/120（2groups） | 120 | 59/61/62/58 | 57/63 | 49 ±5.4/51 ±6.3 | 53 ±5.2 | 2000 IU/d vitamin D3/1000 IU/d vitamin D3 | placebo | 3m | probing depth (PD),plaque index(PI) |
| Effect of systemic antibiotic and  probiotic therapies as adjuvant  treatments of subgingival  instrumentation for periodontitis: a  randomized controlled clinical study | Tatiane Caroline de Souza RAMOS | 2022 | RCT | Brasil | chronic periodontitis(II-III) | Blank | 15/15（2groups） | 15 | 4/11/8/7 | 4/11 | 49.60±7.548/42.20±7.44 | 51.67±5.53 | Lactobacillus reuteri/500 mg  amoxicillin + 400 mg metronidazole | placebo | 90d | bleeding on probing(BOP),plaque index(PI),probing depth (PD),Clinical attachment loss(CAL) |
| Effectiveness of a nutraceutical agent in the non-surgical periodontal therapy: a randomized, controlled clinical trial | Gaetano Isola | 2020 | RCT | Italy | chronic periodontitis(I-III) | Blank | 33 | 33 | 34/32 | Not metioned | 47.8(34-65) | Not metioned | SRP + nutraceutical agent | SRP | 6m | Clinical attachment level(CAL),bleeding on probing(BOP),plaque index(PI) |
| Effectiveness of CoQ10 Oral Supplements  as an Adjunct to Scaling and Root Planing  in Improving Periodontal Health | Sathish Manthena | 2015 | RCT | India | chronic periodontitis(II-III) | Blank | 15 | 15 | 14/16 | Not metioned | 30.05 ± 11.00 | 37.6 ± 13.63 | oral CoQ10 +SRP | placebo +SRP | 1m | plaque index(PI), Gingival index(GI),probing depth(PD) |
| Effectiveness of sub gingival irrigation of an indigenous 1% curcumin  solution on clinical and microbiological parameters in chronic periodontitis  patients: A pilot randomized clinical trial | Sruthima N. V. S | 2013 | RCT | India | chronic periodontitis(II-III) | Blank | 23sites/23sites | 23sites | 12/14 | Not metioned | 30-55 | Not metioned | 1% curcumin (CU)/0.2% chlorhexidine (CHX) | placebo | 3m,6m | bleeding on probing(BOP), plaque index(PI), probing pocket depth(PPD) |
| Effects of Chicory Leaf Extract on Serum Oxidative Stress Markers, Lipid Profile and Periodontal Status in Patients With Chronic Periodontitis | Hossein Babaei, PharmD | 2018 | RCT | Iran | chronic periodontitis(I-III) | Blank | 20 | 20 | 9/11 | 12/8 | 39.45±11.71 | 42.05 ±15.38 | chicory leaf methanolic extract capsule | placebo | 8w | pocket depth (PD) |
| The effects of ginger supplementation on inflammatory, antioxidant, and periodontal parameters in type 2 diabetes mellitus patients with chronic periodontitis under non-surgical periodontal therapy. A double-blind, placebo-controlled trial | Ahmad Zare Javid | 2019 | RCT | Iran | chronic periodontitis(I-III) | type 2 diabetes mellitus | 21 | 21 | 11/10 | 12/9 | 52.81±6.44 | 51.62±5.95 | ginger | placebo | 8w | clinical attachment level (CAL), bleeding on probing (BOP), pocket depth (PD),plaque index(PI) |
| Effects of Lactobacillus reuteri as an adjunct to the treatment of periodontitis in  smokers: randomised clinical trial | L.H. Theodoro | 2019 | RCT | Brazil | chronic periodontitis(I-III) | Blank | 14 | 14 | 5/9 | 10/4 | 47.25±7.10 | 45.07±6.31 | SRP + probiotic tablets | SRP+placebo | 90d | bleeding on probing(BOP), probing depth (PD), Clinical attachment level(CAL) |
| The efects of melatonin supplementation in adjunct with non‑surgical  periodontal therapy on periodontal status, serum melatonin  and infammatory markers in type 2 diabetes mellitus patients  with chronic periodontitis: a double‑blind, placebo‑controlled trial | Hadi Bazyar | 2019 | RCT | Iran | chronic periodontitis(I-III) | type 2 diabetes mellitus | 22 | 22 | 8/14 | 6/16 | 53.72±6.68 | 51.45±5.03 | melatonin | placebo | 8w | clinical attachment level(CAL), pocket depth (PD), bleeding on probing(BOP),plaque index(PI) |
| Effects of natural herb-containing dental toothpaste on periodontal  pathogenic bacteria and clinical parameters: A randomized clinical trial | Satoshi Shirakawa | 2021 | RCT | Japan | chronic periodontitis(I-III) | Blank | 34 | 37 | 8/26 | 8/29 | 62.57 ± 11.16 | 62.4 ± 10.8 | (rhatany  tincture (1.25%), chamomile tincture (1.25%), and myrrh tincture (0.62%)) | placebo | 4w | Gingival index(GI), bleeding on probing(BOP), and probing depth (PD) |
| Effects of Vitamin-B Complex Supplementation on Periodontal Wound Healing | Rodrigo F. Neiva | 2005 | RCT | US | chronic periodontitis(II-III) | Blank | 15 | 15 | 7/8 | 6/9 | 46.2 (38-65) | 47.9 (39-62) | Vit-B capsule | placebo | 90d | Clinical attachment level(CAL),bleeding on probing(BOP), Gingival index(GI),and plaque index(PI) |
| Efficacy and Viability of Subgingival Application of Probiotics as an Adjunct to Scaling and Root Planing in Periodontitis | Suneeta Panicker | 2024 | RCT | India | chronic periodontitis(II-III) | Blank | 31sites | 31sites | Not metioned | | 25-50 | Not metioned | SRP +probiotic paste | SRP | 12w | Gingival index(GI),Probing pocket depth (PPD) |
| Efficacy of a local‑drug delivery gel containing extracts of Quercus  brantii and Coriandrum sativum as an adjunct to scaling and root  planing in moderate chronic periodontitis patients | Jaber Yaghini | 2014 | RCT | Iran | chronic periodontitis(II-III) | Blank | 36sites | 38sites | Not metioned | | 31-52 | Not metioned | SRP+herbal gel | SRP+placebo | 3m | Periodontal pocket depth(PPD), Clinical attachment level(CAL),plaque index(PI) |
| Efficacy of an herbal antioxidant as  an adjunct to nonsurgical periodontal  therapy on procalcitonin levels in  smokers with chronic periodontitis | Sravya M. V. N | 2019 | RCT | India | chronic periodontitis(I-III) | Blank | 20 | 20 | Not metioned | | 35-60 | Not metioned | SRP + Oxitard | SRP | 3m | Gingival index(GI); Probing pocket depth(PPD); Clinical attachment level(CAL) |
| Efficacy of Cucurmin and Aloevera Extracts Gel as Local Drugs  Delivery Agents | Raktapratim Borgohain | 2023 | RCT | India | chronic periodontitis(II-III) | Blank | 10sites/10sites | 10sites | 4/6 | Not metioned | 47.4 | Not metioned | SRP+ curcumin gel/SRP + pure aloe vera gel | SRP | 30d | probing pocket depth(PPD）, Clinical attachment level(CAL), plaque index(PI), Gingival index(GI) |
| Efficacy of Curcumin as an Adjunct to Scaling and Root  Planing in Chronic Periodontitis Patients: A Randomized  Controlled Clinical Trial | Kepu V Raghava | 2019 | RCT | India | chronic periodontitis(I-III) | Blank | 10sites | 10sites | Not metioned | | 25-40 | Not metioned | SRP + curcumin | SRP | 4w | plaque index(PI), Gingival index(GI), probing pocket depth(PPD), and Clinical attachment level(CAL) |
| Efficacy of Curcumin Gel on Zinc, Magnesium, Copper, IL-1β, and TNF-α in Chronic Periodontitis Patients | Chenar Anwar Mohammad | 2020 | RCT | Iraq | chronic periodontitis(I-III) | Blank | 30/30 | 30 | 22/38 | 13/17 | 36.73 ± 6.22 | 37.30 ± 7.08 | SRP+ curcumin gel /SRP alone | placebo | 1m | plaque index(PI), Gingival index(GI),bleeding on probing(BOP), pocket depth（PD）, and clinical attachment level（CAL） |
| Efficacy of grape seed extract gel in the treatment of chronic  periodontitis: A randomized clinical study | Mohammad Rayyan | 2018 | RCT | Saudi Arabia | chronic periodontitis(I-III) | Blank | 48 | 38 | Not metioned | | 43.5 ± 7.9 | Not metioned | formulated 2% mucoadhesive GSE gel | placebo | 4w | pocket depth(PD), Gingival index(GI), plaque index(PI), bleeding on probing(BOP) |
| Efficacy of local drug delivery of  Achyranthes aspera gel in the  management of chronic periodontitis:  A clinical study | Ramanarayana Boyapati | 2017 | RCT | India | chronic periodontitis(I-III) | Blank | 15 | 15 | Not metioned | | Not metioned | | SRP+A. aspera gel | SRP+ placebo | 3m | Gingival index(GI), bleeding on probing(BOP), probing pocket depth（PPD）, and Clinical attachment level(CAL) |
| Efficacy of Pluronic F-127 gel containing green tea  catechin extract on chronic periodontitis – A clinical study | Raghavendra Reddy Nagate | 2020 | RCT | Saudi Arabia | chronic periodontitis(I-III) | Blank | 20sites | 20sites | Not metioned | | 20-40 | Not metioned | SRP+pluronic F-127 gel  containing green tea catechin | SRP | 28d | plaque index(PI), Gingival index(GI), and probing pocket depth (PPD) |
| Efficacy of Systemic Administration  of Alpha Lipoic Acid and Scaling and  Root Planning in Patients with Chronic  Periodontitis and Type 2 Diabetes  Mellitus-A Randomised Controlled Trial | Kinnera Surapaneni | 2018 | RCT | India | chronic periodontitis(I-III) | type 2 diabetes mellitus | 20 | 20 | 18/22 | Not metioned | 35-60 | Not metioned | ALA+SRP | SRP | 3m | Gingival index(GI), Probing Pocket Depth (PPD), and Clinical attachment loss(CAL) |
| Evaluating clinical and laboratory  effects of ozone in non-surgical  periodontal treatment: a randomized  controlled trial | Eltas SEYDANUR DENGIZEK | 2019 | RCT | Turkey | chronic periodontitis(II) | Blank | 19 | 18 | 11/8 | 10/8 | 44.7±5.1 | 45.8±5.6 | SRP+gaseous ozone | SRP+placebo | 1m | plaque index(PI), Gingival index(GI), probing depth(PD）, and Clinical attachment level(CAL). |
| Evaluation of biodegradable gel containing flax seed extract (Linum usitatissimum) as a targeted drug delivery for management of chronic periodontitis | Pappu R | 2019 | RCT | India | chronic periodontitis(I-III) | Blank | 20/20 | 20 | 25/35 | Not metioned | Not metioned | | formulated flax seed extract gel/ flurbiprofen gel | placebo | 3m | plaque index(PI),Gingival index(GI),Probing pocket depth (PPD) and Clinical attachment level(CAL) |
| Evaluation of dietary supplementation  of omega‑3 polyunsaturated fatty  acids as an adjunct to scaling and  root planing on salivary interleukin‑1β levels in patients with chronic  periodontitis: A clinico‑immunological  study | Vanali Vinodbhai Umrania | 2017 | RCT | India | chronic periodontitis(I-III) | Blank | 20 | 20 | 12/8 | 13/7 | 43.5 ± 5.8 | 44 ± 6.44 | ω‑3 PUFA+SRP | SRP | 3m | plaque index(PI), Gingival index(GI), pocket depth(PD) and Clinical attachment level(CAL) |
| Evaluation of Grape Seed Formulation as an  Adjunct to Scaling and Root Planing on Oxidative  Stress, Inflammatory Status and Glycaemic  Control in Type 2 Diabetic Patients with Chronic  Periodontitis: A Randomised Controlled Trial | Sameekshya Acharya | 2021 | RCT | India | chronic periodontitis(I-III) | type 2 diabetes mellitus | 24 | 24 | Not metioned | | 30-60 | Not metioned | SRP+grape seed  formulation | SRP+placebo | 3m,6m | plaque index(PI), Pocket Probing Depth (PPD) and Clinical attachment level(CAL) |
| Evaluation of superoxide dismutase levels in  local drug delivery system containing 0.2%  curcumin strip as an adjunct to scaling and  root planing in chronic periodontitis: A clinical  and biochemical study | Sugumari Elavarasu | 2016 | RCT | India | chronic periodontitis(II) | Blank | 15sites/15sites | 5sites | Not metioned | | 35-55 | Not metioned | SRP +placement of curcumin strip | SRP | 21d | plaque index(PI), Gingival index(GI),probing pocket depth(PPD) |
| Evaluation of the efficacy of lycopene gel compared with minocycline hydrochloride microspheres as an adjunct to nonsurgical periodontal treatment: A randomised clinical trial | Aya Ali | 2021 | RCT | Iraq | chronic periodontitis(I-III) | Blank | 23sites | 23sites/23sites | 15/8 | Not metioned | 45.7±11.5 | Not metioned | lycopene (Lyc) antioxidant gel | placebo gel | 30d | plaque index(PI), bleeding on probing(BOP), probing pocket depth（PPD）, and clinical attachment level（CAL） |
| Evaluation of Turmeric Chip Compared with Chlorhexidine Chip as a Local Drug Delivery Agent in the Treatment of Chronic Periodontitis: A Split Mouth Randomized Controlled Clinical Trial | Abhilasha Singh | 2018 | RCT | India | chronic periodontitis(I-III) | Blank | 40sites/40sites | 40sites | 22/18 | Not metioned | 34(30–50) | Not metioned | turmeric chip+SRP/CHX gluconate chip+SRP | SRP | 3m | plaque index(PI), Gingival index(GI), probing pocket depth(PPD) |
| Green Tea Intake as an Adjunct to Mechanical  Periodontal Therapy for the Management of Mild to  Moderate Chronic Periodontitis: A Randomised  Controlled Clinical Trial | Aditi Chopra | 2016 | RCT | India | chronic periodontitis(II-III) | Blank | 56 | 59 | 31/25 | 30/29 | 35.60 ± 3.78 | 36.70 ± 5.30 | SRP+green tea supplements | SRP+placebo | 3m | Gingival index(GI), plaque index(PI), clinical attachment level（CAL）, bleeding on probing(BOP),and probing depth（PPD） |
| Herbal anti-inflammatory  immunomodulators as host  modulators in chronic periodontitis  patients: a randomised, double-blind,  placebo-controlled, clinical trial | Girish D. Deore | 2014 | RCT | India | chronic periodontitis(II-III) | Blank | 30 | 30 | 31/29 | Not metioned | 46.47±6.01 | 45.00±4.91 | SRP+Septilin | SRP+placebo | 6w | plaque index(PI),Gingival index(GI),Pocket depth（PD）,Clinical attachment level(CAL) |
| Impact of a Specific Collagen Peptide Food Supplement on Periodontal Inflammation in Aftercare Patients—A Randomised Controlled Trial | Yvonne Jockel-Schneider | 2022 | RCT | Germany | chronic periodontitis(I-II) | Blank | 20 | 19 | 6/14 | 12/7 | 59.6 (56.6–62.6) | 59.6(55.2–63.1) | professional mechanical plaque removal (PMPR)+specific collagen peptide preparation | PMPR+placebo | 90d | bleeding on probing(BOP),Gingival index(GI), probing pocket depth (PPD) |
| Impact of Cranberry Juice Enriched with Omega-3 Fatty Acids Adjunct with Nonsurgical Periodontal Treatment on Metabolic Control and Periodontal Status in Type 2 Patients with Diabetes with Periodontal Disease | Ahmad Zare Javid | 2018 | RCT | Iran | chronic periodontitis(I-III) | type 2 diabetes mellitus | 10/9/10（3groups） | 12 | 5/5/5/4/2/8 | 2/10 | 57.75 ± 8.58/57.88 ± 6.03/ 53.14±6.91 | 53.60 ± 6.23 | receiving omega-3/cranberry juice/cranberry juice enriched with omega-3 | Placebo | 8w | probing depth(PD） |
| Impact of green tea intake on clinical improvement in chronic periodontitis: A randomized clinical trial | F. Taleghani | 2018 | RCT | Iran | chronic periodontitis(I-III) | Blank | 15 | 15 | 5/10 | 5/10 | Not metioned | | SRP+green tea | SRP | 6w | Pocket depth(PD),plaque index(PI) |
| Impact of resveratrol supplementation on inflammatory, antioxidant, and  periodontal markers in Type 2 Diabetic Patients with Chronic Periodontitis | Ahmad Zare Javid | 2019 | RCT | Iran | chronic periodontitis(I-III) | type 2 diabetes mellitus | 25 | 25 | Not metioned | | 30-60 | Not metioned | resveratrol supplements | placebo | 4w | clinical attachment level (CAL) |
| The Impacts of Synbiotic Supplementation on Periodontal Indices and Biomarkers of Oxidative Stress in Type 2 Diabetes Mellitus Patients with Chronic Periodontitis Under Non-Surgical Periodontal Therapy. A Double-Blind, Placebo-Controlled Trial | Hadi Bazyar | 2020 | RCT | Iran | chronic periodontitis(I-III) | type 2 diabetes mellitus | 23 | 24 | 6/17 | 8/16 | 48.6 ± 5.8 | 50.1 ± 3.6 | multispecies probiotic supplement plus 100 mg fructo-oligosaccharide | placebo | 8w | clinical attachment level(CAL), pocket depth (PD), bleeding on probing(BOP),plaque index(PI) |
| Improvement in periodontal healing  after periodontal surgery supported  by nutritional supplement drinks | Jaeri Lee | 2014 | RCT | Korea | chronic periodontitis(III) | Blank | 14 | 9 | Not metioned | | 51.1(42–59) | 46.9(40–54) | nutritional supplement drinks | placebo | 8w | Gingival index(GI) |
| Influence of Vitamin D & Calcium  Supplementation in the Management of  Periodontitis | JAYACHANDRAN Perayil | 2015 | RCT | India | chronic periodontitis(II) | Blank | 36 | 41 | Not metioned | | 35-55 | Not metioned | vitamin D (250IU/ day) and calcium (500 mg/day) supplementation | placebo | 3m | Gingival index(GI), pocket probing depth(PPD), clinical attachment level(CAL) |
| Intrapocket application of tea tree oil gel  in the treatment of stage 2 periodontitis | Maha R. Taalab | 2021 | RCT | Egypt | chronic periodontitis(II) | Blank | 15 | 15 | 5/10 | 5/10 | 30.5±5.6 | 28.9±6.3 | SRP+locally delivered 5% Melaleuca alternifolia(TTO) gel | SRP | 3m,6m | pocket probing depth (PPD), clinical attachment level(CAL), Gingival index(GI) and bleeding on probing(BOP) |
| Is dietary melatonin supplementation a viable adjunctive  therapy for chronic periodontitis?—A randomized controlled  clinical trial | Hesham El-Sharkawy | 2019 | RCT | Egypt | chronic periodontitis(I) | Blank | 38 | 36 | 21/17 | 20/16 | 45.6 ± 7.1 | 46.7 ± 8.3 | SRP+ melatonin capsule | SRP+placebo | 3m,6m | Clinical attachment level(CAL) pocket depth (PD), bleeding on probing(BOP) |
| Lycopene solid lipid microparticles with enhanced effect on gingival crevicular fluid protein carbonyl as a biomarker of oxidative stress in patients with chronic periodontitis | Maie S. Tawfik | 2019 | RCT | Egypt | chronic periodontitis(II-III) | Blank | 8/8（2groups） | 8 | 5/11 | Not metioned | 43±6.4 | Not metioned | SRP + lycopene loaded solid lipid microparticles(SLMs) | SRP | 1m | plaque index(PI),pocket depth (PD), clinical attachment level(CAL),Gingival index(GI) |
| Nonsurgical Treatment of Periodontitis in Menopausal Patients: A Randomized Control Trial | Hadir F. Eldessouky | 2024 | RCT | Egypt | chronic periodontitis(II-III) | Blank | 10 | 10 | 0/20 | Not metioned | 45-55 | Not metioned | polyunsaturated fatty acids(PUFAs) | placebo | 6m | plaque index(PI), Gingival index(GI), probing pocket depth(PPD),clinical attachment level(CAL) |
| Omega-3 Polyunsaturated Fatty Acids EPA and DHA as an Adjunct to Non-Surgical Treatment of Periodontitis: A Randomized Clinical Trial | Mirella Sta ´ndo | 2020 | RCT | Poland | chronic periodontitis(III) | Blank | 20 | 20 | 10/10 | 9/11 | 47.5 ± 9.63 | 49.3 ± 12.80 | SRP+ dietary fish oil (FO) | SRP | 3m | plaque index(PI) , bleeding on probing(BOP), probing depth(PD) |
| A randomized clinical trial to evaluate  and compare the efficacy of triphala  mouthwash with 0.2% chlorhexidine  in hospitalized patients with  periodontal diseases | Ritam S. Naiktari | 2014 | RCT | India | chronic periodontitis(I-III) | Blank | 40/40（2groups） | 40 | 78/42 | Not metioned | Not metioned | | triphala  mouthwash/0.2% chlorhexidine | placebo | 15d | plaque index(PI), Gingival index(GI) |
| A randomized, double-blind, placebo controlled multicenter study for evaluating the effects of fixed-dose combinations of vitamin C, vitamin E, lysozyme, and carbazochrome on gingival inflammation in chronic periodontitis patients | Ji-Youn Hong | 2019 | RCT | Korea | chronic periodontitis(II-III) | Blank | 48 | 49 | 19/29 | 16/33 | 37.83 ± 12.72 | 43.02 ± 14.30 | combinations of vitamin C, vitamin E, lysozyme and carbazochrome (CELC) | placebo | 8w | Gingival index(GI),plaque index(PI), probing depth(PD), Clinical attachment level(CAL) |
| Semelil as Adjunctive Therapy in Chronic Periodontitis: A  Preliminary Randomized Controlled Clinical Study | Hoori Aslroosta | 2021 | RCT | Iran | chronic periodontitis(II-III) | Blank | 15 | 10 | 12/13 | Not metioned | Not metioned | | SRP+Semelil | SRP+placebo | 3m | probing depth(PD), Clinical attachment level(CAL)，Gingival index(GI), plaque index(PI) |
| Study on the Effects of Melatonin on Glycemic Control and Periodontal Parameters in Patients with Type II Diabetes Mellitus and Periodontal Disease | Diana-Maria Anton | 2021 | RCT | Romania | chronic periodontitis(I-III) | type 2 diabetes mellitus | 25 | 25 | 14/11 | 15/10 | 53.24 ± 3.4 | 52.21 ± 3.1 | SRP+ melatonin | SRP+placebo | 8w | probing depth(PD); clinical attachment level(CAL);bleeding on probing(BOP) |
| Sustained Release of Liposomal Curcumin: Enhanced Periodontal Outcomes in Diabetic Patients | Abdallah Khalil, A. | 2024 | RCT | Egypt | chronic periodontitis(II) | type 2 diabetes mellitus | 10/10(2groups） | 10 | Not metioned | | 35-60 | Not metioned | SRP+ curcumin gel | SRP+placebo | 12w | plaque index(PI), Gingival index(GI), probing depth (PD), Clinical attachment level(CAL) |
| Therapeutic Potential of Melatonin in Periodontitis: A Randomised, Placebo  Controlled, Double Blind Study. | Marawar AP | 2014 | RCT | India | chronic periodontitis(I-III) | Blank | 80 | 80 | Not metioned | | Not metioned | Not metioned | SRP+melatonin | SRP+placebo | 90d | Gingival index(GI) |
| To Evaluate the Effect of Vitamin B Complex on Wound Healing – A  Clinical and Microbiological Study | Rupali Kalsi | 2024 | RCT | India | chronic periodontitis(I-III) | Blank | 10sites | 10sites | Not metioned | | 25–50 | Not metioned | vitamin B | placebo | 90d | plaque index(PI), Gingival index(GI),bleeding on probing(BOP) |
| Using Ginger Supplement in Adjunct with Non-surgical Periodontal Therapy Improves  Metabolic and Periodontal Parameters in Patients with Type 2 Diabetes Mellitus (DM) and  Chronic Periodontitis. A Double-Blind, Placebo-Controlled Trial | Hasan Gholinezhad | 2020 | RCT | Iran | chronic periodontitis(I-III) | type 2 diabetes mellitus | 21 | 21 | 10/11 | 9/12 | 52.81 ± 6.44 | 51.62 ± 5.95 | ginger | placebo | 8w | clinical attachment level(CAL), Pocket depth (PD), plaque index(PI),bleeding on probing(BOP) |

**Table S4** NMA results for the decline in BOP across interventions.

| placebo |  |  |  |  |  |  |  |  |
| --- | --- | --- | --- | --- | --- | --- | --- | --- |
| 12.14 (-7.74, 32.18) | multinutrient |  |  |  |  |  |  |  |
| 5.11 (-4.12, 14.36) | -7.02 (-29.1, 15) | probiotic |  |  |  |  |  |  |
| 8.99 (-18.53, 36.36) | -3.14 (-37.02, 30.58) | 3.9 (-24.98, 32.75) | melatonin |  |  |  |  |  |
| 2.62 (-23.96, 29.08) | -9.51 (-43, 23.51) | -2.49 (-30.6, 25.46) | -6.34 (-44.59, 31.84) | antioxidant |  |  |  |  |
| 16.06 (-10.27, 42.35) | 3.97 (-29.39, 36.8) | 10.95 (-16.92, 38.83) | 7.07 (-30.82, 45.19) | 13.48 (-23.83, 50.63) | her-anti |  |  |  |
| 3.09 (-26.03, 32.25) | -9.04 (-44.31, 26.15) | -2.03 (-32.51, 28.5) | -5.93 (-45.89, 34.06) | 0.47 (-38.83, 40.06) | -12.94 (-52.25, 26.55) | VC |  |  |
| 4.06 (-22.75, 30.96) | -8.09 (-41.7, 25.27) | -1.04 (-29.47, 27.32) | -4.94 (-43.41, 33.7) | 1.44 (-36.2, 39.42) | -11.97 (-49.62, 25.59) | 0.93 (-38.44, 40.59) | VB |  |
| -3.01 (-30.15, 24.28) | -15.12 (-48.87, 18.53) | -8.13 (-36.73, 20.7) | -12.03 (-50.48, 26.44) | -5.65 (-43.38, 32.43) | -19.07 (-56.78, 18.71) | -6.1 (-45.85, 33.58) | -7.05 (-45.2, 31.02) | \|  \| \| --- \| \| VD \| \|  \| |

NOTE: RR with 95% CIs in parentheses. Bold values denote a statistically significant difference.

her-anti, herbal extract(antioxidants)

**Table S5** NMA results for the decline in CAL (3m) across interventions.

| plcabo |  |  |  |  |  |  |  |  |  |  |  |  |  |  |
| --- | --- | --- | --- | --- | --- | --- | --- | --- | --- | --- | --- | --- | --- | --- |
| 0.03 (-0.87, 0.94) | multinutrient |  |  |  |  |  |  |  |  |  |  |  |  |  |
| 0.11 (-0.45, 0.66) | 0.07 (-0.99, 1.14) | probiotic |  |  |  |  |  |  |  |  |  |  |  |  |
| 0.98 (0.29, 1.68) | 0.95 (-0.19, 2.09) | 0.87 (-0.01, 1.77) | melatonin |  |  |  |  |  |  |  |  |  |  |  |
| 0.04 (-1.02, 1.09) | 0.01 (-1.39, 1.39) | -0.06 (-1.27, 1.12) | -0.94 (-2.22, 0.31) | mineral |  |  |  |  |  |  |  |  |  |  |
| 0.22 (-0.39, 0.85) | 0.19 (-0.91, 1.29) | 0.12 (-0.71, 0.95) | -0.76 (-1.69, 0.17) | 0.18 (-1.03, 1.42) | antioxidant |  |  |  |  |  |  |  |  |  |
| 0.88 (-1.09, 2.89) | 0.85 (-1.32, 3.04) | 0.77 (-1.28, 2.84) | -0.1 (-2.2, 2.01) | 0.84 (-1.38, 3.11) | 0.65 (-1.41, 2.75) | mel+SRP |  |  |  |  |  |  |  |  |
| 0.64 (-0.88, 2.17) | 0.6 (-1.16, 2.38) | 0.53 (-1.09, 2.16) | -0.34 (-2.02, 1.34) | 0.59 (-1.25, 2.46) | 0.41 (-1.23, 2.06) | -0.25 (-1.66, 1.17) | anti+SRP |  |  |  |  |  |  |  |
| -0.01 (-1.47, 1.45) | -0.05 (-1.76, 1.68) | -0.12 (-1.68, 1.44) | -1 (-2.61, 0.63) | -0.06 (-1.85, 1.76) | -0.24 (-1.83, 1.35) | -0.9 (-2.25, 0.45) | **-0.65 (-1.09, -0.22)** | SRP |  |  |  |  |  |  |
| 0.43 (0.02, 0.84) | 0.39 (-0.61, 1.39) | 0.32 (-0.37, 1.01) | -0.55 (-1.37, 0.25) | 0.38 (-0.74, 1.53) | 0.2 (-0.55, 0.95) | -0.45 (-2.41, 1.49) | -0.21 (-1.68, 1.25) | 0.44 (-0.96, 1.84) | her-anti |  |  |  |  |  |
| 0.19 (-1.16, 1.54) | 0.15 (-1.47, 1.79) | 0.08 (-1.38, 1.54) | -0.79 (-2.31, 0.71) | 0.14 (-1.56, 1.87) | -0.04 (-1.52, 1.44) | -0.7 (-3.11, 1.71) | -0.45 (-2.5, 1.59) | 0.21 (-1.79, 2.2) | -0.24 (-1.66, 1.17) | her-min |  |  |  |  |
| 0.43 (-1.1, 1.97) | 0.4 (-1.38, 2.19) | 0.32 (-1.3, 1.96) | -0.55 (-2.23, 1.14) | 0.39 (-1.46, 2.27) | 0.21 (-1.45, 1.87) | -0.45 (-1.88, 0.98) | -0.2 (-0.85, 0.44) | 0.45 (-0.02, 0.92) | 0.01 (-1.47, 1.49) | 0.24 (-1.8, 2.3) | her-anti+  SRP |  |  |  |
| 0.02 (-1.33, 1.38) | -0.01 (-1.64, 1.62) | -0.08 (-1.55, 1.37) | -0.96 (-2.48, 0.57) | -0.02 (-1.72, 1.71) | -0.2 (-1.69, 1.28) | -0.86 (-3.28, 1.54) | -0.61 (-2.66, 1.42) | 0.04 (-1.96, 2.03) | -0.4 (-1.82, 1.01) | -0.17 (-2.08, 1.75) | -0.4 (-2.47, 1.63) | VC |  |  |
| 0.43 (-1.04, 1.9) | 0.4 (-1.34, 2.12) | 0.32 (-1.25, 1.89) | -0.55 (-2.18, 1.07) | 0.39 (-1.41, 2.2) | 0.2 (-1.39, 1.79) | -0.45 (-2.93, 2.01) | -0.21 (-2.33, 1.91) | 0.44 (-1.63, 2.52) | 0 (-1.53, 1.53) | 0.24 (-1.75, 2.24) | -0.01 (-2.13, 2.12) | 0.4 (-1.59, 2.41) | VB |  |
| 1.23 (0.32, 2.16) | 1.2 (-0.09, 2.5) | 1.12 (0.05, 2.21) | 0.25 (-0.9, 1.4) | 1.19 (-0.2, 2.6) | 1.01 (-0.1, 2.12) | 0.34 (-1.85, 2.54) | 0.59 (-1.18, 2.38) | 1.25 (-0.48, 2.97) | 0.8 (-0.2, 1.82) | 1.04 (-0.58, 2.68) | 0.8 (-0.99, 2.58) | 1.21 (-0.42, 2.84) | \| 0.8 (-0.92, 2.54) \| \| --- \| | VD |

NOTE: RR with 95% CIs in parentheses. Bold values denote a statistically significant difference.

her-anti, herbal extract(antioxidants); her-min, herbal extract(mineral); anti, antioxidants; SRP, scaling and root planing.

**Table S6** NMA results for the decline in CAL (6m) across interventions.

| placebo |  |  |  |  |
| --- | --- | --- | --- | --- |
| -0.3 (-1.54, 0.94) | probiotic |  |  |  |
| 0.91 (-1.67, 3.61) | 1.21 (-1.64, 4.17) | anti+SRP |  |  |
| 0.28 (-2.08, 2.64) | 0.58 (-2.08, 3.23) | -0.63 (-1.86, 0.49) | SRP |  |
| 0.6 (-1.03, 2.22) | 0.9 (-1.14, 2.93) | -0.31 (-2.44, 1.71) | 0.32 (-1.4, 2.03) | \| her-anti \| \| --- \| |

NOTE: RR with 95% CIs in parentheses. Bold values denote a statistically significant difference.

her-anti, herbal extract(antioxidants); anti, antioxidants; SRP, scaling and root planing.

**Table S7** NMA results for the decline in GI across interventions.

| plcabo |  |  |  |  |  |  |  |  |  |  |  |  |  |  |  |
| --- | --- | --- | --- | --- | --- | --- | --- | --- | --- | --- | --- | --- | --- | --- | --- |
| 0.43 (-0.11, 0.98) | multinutrient |  |  |  |  |  |  |  |  |  |  |  |  |  |  |
| 0.04 (-0.49, 0.57) | -0.39 (-1.15, 0.37) | probiotic |  |  |  |  |  |  |  |  |  |  |  |  |  |
| 0.38 (-0.16, 0.98) | -0.05 (-0.81, 0.76) | 0.34 (-0.41, 1.14) | melatonin |  |  |  |  |  |  |  |  |  |  |  |  |
| 0.79 (0.03, 1.55) | 0.36 (-0.58, 1.29) | 0.75 (-0.18, 1.67) | 0.41 (-0.56, 1.32) | mineral |  |  |  |  |  |  |  |  |  |  |  |
| -0.05 (-0.49, 0.36) | -0.49 (-1.19, 0.19) | -0.1 (-0.79, 0.57) | -0.43 (-1.19, 0.24) | -0.84 (-1.72, 0.02) | antioxidant |  |  |  |  |  |  |  |  |  |  |
| 0.96 (-0.06, 1.99) | 0.53 (-0.64, 1.69) | 0.92 (-0.24, 2.08) | 0.58 (-0.62, 1.73) | 0.17 (-1.1, 1.45) | 1.02 (0.01, 2.04) | pro+SRP |  |  |  |  |  |  |  |  |  |
| 0.58 (-0.4, 1.56) | 0.15 (-0.98, 1.26) | 0.54 (-0.57, 1.66) | 0.2 (-0.96, 1.3) | -0.21 (-1.45, 1.03) | 0.64 (-0.31, 1.61) | -0.38 (-1.5, 0.73) | mel+SRP |  |  |  |  |  |  |  |  |
| 0.49 (-0.21, 1.19) | 0.05 (-0.84, 0.94) | 0.44 (-0.44, 1.32) | 0.11 (-0.82, 0.97) | -0.3 (-1.33, 0.73) | 0.54 (-0.13, 1.23) | -0.48 (-1.36, 0.4) | -0.1 (-0.92, 0.73) | anti+SRP |  |  |  |  |  |  |  |
| 0.12 (-0.5, 0.74) | -0.31 (-1.14, 0.51) | 0.08 (-0.74, 0.89) | -0.26 (-1.13, 0.55) | -0.67 (-1.65, 0.31) | 0.17 (-0.41, 0.78) | -0.84 (-1.66, -0.02) | -0.46 (-1.22, 0.3) | -0.36 (-0.69, -0.04) | SRP |  |  |  |  |  |  |
| 0.55 (0.28, 0.83) | 0.12 (-0.5, 0.73) | 0.51 (-0.09, 1.1) | 0.17 (-0.49, 0.77) | -0.24 (-1.04, 0.57) | 0.61 (0.13, 1.1) | -0.41 (-1.44, 0.62) | -0.03 (-1.01, 0.95) | 0.07 (-0.63, 0.76) | 0.43 (-0.18, 1.05) | her-anti |  |  |  |  |  |
| 0.19 (-0.58, 0.97) | -0.24 (-1.2, 0.7) | 0.15 (-0.79, 1.09) | -0.19 (-1.18, 0.74) | -0.6 (-1.69, 0.48) | 0.24 (-0.63, 1.14) | -0.77 (-2.06, 0.51) | -0.39 (-1.64, 0.86) | -0.3 (-1.34, 0.75) | 0.07 (-0.92, 1.06) | -0.36 (-1.18, 0.47) | her-min |  |  |  |  |
| 0.77 (-0.26, 1.79) | 0.34 (-0.83, 1.49) | 0.73 (-0.43, 1.88) | 0.39 (-0.81, 1.53) | -0.02 (-1.3, 1.25) | 0.83 (-0.18, 1.84) | -0.19 (-1.35, 0.96) | 0.19 (-0.92, 1.3) | 0.29 (-0.59, 1.16) | 0.65 (-0.17, 1.47) | 0.22 (-0.8, 1.24) | 0.58 (-0.71, 1.86) | her-min  +SRP |  |  |  |
| 0.49 (-0.18, 1.15) | 0.05 (-0.81, 0.91) | 0.44 (-0.41, 1.29) | 0.11 (-0.8, 0.95) | -0.3 (-1.31, 0.7) | 0.54 (-0.09, 1.19) | -0.48 (-1.33, 0.37) | -0.09 (-0.89, 0.7) | 0 (-0.4, 0.4) | 0.36 (0.12, 0.6) | -0.06 (-0.73, 0.6) | 0.3 (-0.73, 1.31) | -0.29 (-1.14, 0.56) | her-anti  +SRP |  |  |
| 0.13 (-0.72, 0.98) | -0.3 (-1.31, 0.7) | 0.09 (-0.91, 1.08) | -0.25 (-1.29, 0.74) | -0.66 (-1.8, 0.48) | 0.18 (-0.76, 1.14) | -0.83 (-2.17, 0.5) | -0.45 (-1.74, 0.85) | -0.35 (-1.46, 0.74) | 0.01 (-1.04, 1.06) | -0.42 (-1.31, 0.47) | -0.06 (-1.21, 1.09) | -0.64 (-1.97, 0.69) | -0.36 (-1.44, 0.72) | VC |  |
| -0.03 (-0.79, 0.73) | -0.46 (-1.4, 0.47) | -0.07 (-1, 0.85) | -0.41 (-1.38, 0.5) | -0.82 (-1.9, 0.25) | 0.02 (-0.84, 0.9) | -0.99 (-2.27, 0.29) | -0.61 (-1.85, 0.62) | -0.52 (-1.55, 0.51) | -0.15 (-1.13, 0.83) | -0.58 (-1.39, 0.23) | -0.22 (-1.31, 0.86) | -0.8 (-2.08, 0.48) | -0.52 (-1.52, 0.49) | -0.16 (-1.3, 0.98) | VB |

NOTE: RR with 95% CIs in parentheses. Bold values denote a statistically significant difference.

her-anti, herbal extracts(antioxidants); her-min, herbal extracts(mineral); pro,probiotics;mel,melatonin;anti,antioxidants;SRP,scaling and root planing.

**Table S8** NMA results for the decline in PI across interventions.

| plcabo |  |  |  |  |  |  |  |  |  |  |  |  |  |
| --- | --- | --- | --- | --- | --- | --- | --- | --- | --- | --- | --- | --- | --- |
| -0.08 (-0.49, 0.34) | multinutrient |  |  |  |  |  |  |  |  |  |  |  |  |
| 0 (-0.45, 0.46) | 0.08 (-0.54, 0.7) | probiotic |  |  |  |  |  |  |  |  |  |  |  |
| 0.15 (-0.34, 0.64) | 0.23 (-0.41, 0.87) | 0.14 (-0.52, 0.82) | melatonin |  |  |  |  |  |  |  |  |  |  |
| 0.38 (-0.13, 0.87) | 0.46 (-0.2, 1.1) | 0.38 (-0.31, 1.04) | 0.23 (-0.48, 0.92) | antioxidant |  |  |  |  |  |  |  |  |  |
| 0.25 (-0.8, 1.29) | 0.33 (-0.81, 1.45) | 0.25 (-0.91, 1.38) | 0.1 (-1.06, 1.26) | -0.13 (-1.29, 1.04) | mel+SRP |  |  |  |  |  |  |  |  |
| 0.36 (-0.48, 1.2) | 0.44 (-0.5, 1.38) | 0.36 (-0.61, 1.31) | 0.22 (-0.77, 1.18) | -0.02 (-1, 0.98) | 0.11 (-0.61, 0.84) | anti+SRP |  |  |  |  |  |  |  |
| 0.26 (-0.55, 1.06) | 0.34 (-0.57, 1.24) | 0.26 (-0.67, 1.18) | 0.11 (-0.83, 1.04) | -0.12 (-1.06, 0.84) | 0.01 (-0.67, 0.69) | -0.1 (-0.36, 0.15) | SRP |  |  |  |  |  |  |
| **0.4 (0.17, 0.63)** | 0.48 (0, 0.95) | 0.4 (-0.12, 0.9) | 0.25 (-0.29, 0.78) | 0.02 (-0.52, 0.58) | 0.15 (-0.87, 1.18) | 0.04 (-0.78, 0.85) | 0.14 (-0.63, 0.91) | her-anti |  |  |  |  |  |
| 0.27 (-0.76, 1.3) | 0.35 (-0.77, 1.46) | 0.27 (-0.87, 1.39) | 0.12 (-1.03, 1.26) | -0.11 (-1.25, 1.04) | 0.02 (-0.92, 0.96) | -0.09 (-0.79, 0.61) | 0.01 (-0.64, 0.66) | -0.13 (-1.14, 0.88) | her-min+  SRP |  |  |  |  |
| 0.53 (-0.29, 1.35) | 0.61 (-0.32, 1.52) | 0.53 (-0.42, 1.46) | 0.38 (-0.58, 1.33) | 0.15 (-0.81, 1.12) | 0.28 (-0.42, 0.98) | 0.17 (-0.13, 0.46) | **0.27 (0.09, 0.44)** | 0.13 (-0.66, 0.92) | 0.26 (-0.42, 0.93) | her-anti+  SRP |  |  |  |
| 0.1 (-0.57, 0.77) | 0.18 (-0.62, 0.96) | 0.09 (-0.72, 0.91) | -0.05 (-0.88, 0.78) | -0.28 (-1.1, 0.56) | -0.15 (-1.39, 1.11) | -0.26 (-1.34, 0.82) | -0.16 (-1.2, 0.89) | -0.3 (-1.01, 0.41) | -0.17 (-1.4, 1.07) | -0.43 (-1.49, 0.64) | VC |  |  |
| 0.2 (-0.47, 0.86) | 0.28 (-0.51, 1.06) | 0.2 (-0.62, 1) | 0.05 (-0.78, 0.87) | -0.18 (-1.01, 0.66) | -0.05 (-1.29, 1.2) | -0.16 (-1.23, 0.91) | -0.06 (-1.1, 0.98) | -0.2 (-0.9, 0.5) | -0.07 (-1.29, 1.16) | -0.33 (-1.38, 0.73) | 0.1 (-0.84, 1.04) | VB |  |
| 0.04 (-0.6, 0.68) | 0.12 (-0.65, 0.88) | 0.03 (-0.75, 0.82) | -0.11 (-0.92, 0.69) | -0.34 (-1.15, 0.48) | -0.21 (-1.44, 1.03) | -0.32 (-1.38, 0.74) | -0.22 (-1.24, 0.81) | -0.36 (-1.04, 0.32) | -0.23 (-1.44, 0.99) | -0.49 (-1.53, 0.55) | -0.06 (-0.99, 0.87) | -0.16 (-1.08, 0.76) | VD |

NOTE: RR with 95% CIs in parentheses. Bold values denote a statistically significant difference.

her-anti, herbal extracts(antioxidants); her-min, herbal extracts(mineral); anti, antioxidants; SRP,scaling and root planing.

**Table S9** NMA results for the decline in PPD across interventions.

| plcabo |  |  |  |  |  |  |  |  |  |  |  |  |  |
| --- | --- | --- | --- | --- | --- | --- | --- | --- | --- | --- | --- | --- | --- |
| 0.21 (-0.54, 0.96) | multinutrient |  |  |  |  |  |  |  |  |  |  |  |  |
| 0.11 (-0.34, 0.57) | -0.1 (-0.98, 0.79) | probiotic |  |  |  |  |  |  |  |  |  |  |  |
| 1.11 (0.49, 1.74) | 0.9 (-0.06, 1.89) | 1 (0.23, 1.77) | melatonin |  |  |  |  |  |  |  |  |  |  |
| 0.1 (-0.91, 1.09) | -0.11 (-1.39, 1.14) | -0.02 (-1.13, 1.08) | -1.02 (-2.21, 0.15) | mineral |  |  |  |  |  |  |  |  |  |
| 0.62 (0.16, 1.09) | 0.42 (-0.47, 1.31) | 0.51 (-0.14, 1.16) | -0.49 (-1.27, 0.28) | 0.53 (-0.57, 1.64) | antioxidant |  |  |  |  |  |  |  |  |
| 1.25 (-0.42, 2.91) | 1.04 (-0.79, 2.86) | 1.13 (-0.6, 2.86) | 0.13 (-1.65, 1.9) | 1.15 (-0.79, 3.1) | 0.62 (-1.04, 2.27) | pro+SRP |  |  |  |  |  |  |  |
| 1.11 (-0.07, 2.28) | 0.9 (-0.5, 2.3) | 0.99 (-0.27, 2.25) | -0.01 (-1.34, 1.33) | 1.01 (-0.54, 2.57) | 0.48 (-0.69, 1.65) | -0.14 (-1.61, 1.33) | anti+SRP |  |  |  |  |  |  |
| 0.4 (-0.6, 1.41) | 0.19 (-1.06, 1.45) | 0.29 (-0.82, 1.39) | -0.71 (-1.89, 0.47) | 0.31 (-1.1, 1.74) | -0.22 (-1.21, 0.77) | -0.84 (-2.17, 0.49) | -0.7 (-1.32, -0.08) | SRP |  |  |  |  |  |
| 0.62 (0.23, 1.01) | 0.41 (-0.44, 1.26) | 0.5 (-0.09, 1.1) | -0.5 (-1.23, 0.23) | 0.52 (-0.54, 1.61) | -0.01 (-0.6, 0.59) | -0.63 (-2.29, 1.04) | -0.49 (-1.67, 0.69) | 0.21 (-0.79, 1.22) | her-anti |  |  |  |  |
| 1.26 (-0.44, 2.95) | 1.05 (-0.81, 2.9) | 1.14 (-0.61, 2.89) | 0.14 (-1.67, 1.95) | 1.16 (-0.8, 3.13) | 0.63 (-1.05, 2.31) | 0.01 (-1.89, 1.91) | 0.15 (-1.35, 1.64) | 0.85 (-0.51, 2.21) | 0.64 (-1.05, 2.33) | her-min+  SRP |  |  |  |
| 1.09 (0, 2.16) | 0.88 (-0.44, 2.19) | 0.97 (-0.2, 2.14) | -0.03 (-1.28, 1.21) | 0.99 (-0.47, 2.47) | 0.46 (-0.61, 1.52) | -0.16 (-1.55, 1.22) | -0.02 (-0.76, 0.71) | 0.68 (0.29, 1.07) | 0.47 (-0.62, 1.54) | -0.17 (-1.59, 1.25) | her-anti+  SRP |  |  |
| -0.07 (-1.01, 0.88) | -0.28 (-1.49, 0.93) | -0.18 (-1.24, 0.87) | -1.18 (-2.32, -0.05) | -0.16 (-1.54, 1.23) | -0.69 (-1.75, 0.36) | -1.32 (-3.23, 0.6) | -1.17 (-2.68, 0.34) | -0.47 (-1.85, 0.91) | -0.68 (-1.71, 0.33) | -1.32 (-3.25, 0.61) | -1.15 (-2.59, 0.29) | VB |  |
| 0.86 (-0.06, 1.79) | 0.65 (-0.55, 1.86) | 0.75 (-0.28, 1.78) | -0.25 (-1.37, 0.87) | 0.77 (-0.59, 2.14) | 0.24 (-0.79, 1.28) | -0.38 (-2.29, 1.53) | -0.25 (-1.74, 1.26) | 0.46 (-0.9, 1.82) | 0.24 (-0.75, 1.25) | -0.39 (-2.31, 1.55) | -0.22 (-1.64, 1.2) | 0.93 (-0.39, 2.26) | VD |

NOTE: RR with 95% CIs in parentheses. Bold values denote a statistically significant difference.

her-anti, herbal extracts(antioxidants); her-min, herbal extracts(mineral); anti, antioxidants; SRP,scaling and root planing.
